# Inhibition of ROS and upregulation of inflammatory cytokines by FoxO3a promotes survival against *Salmonella* typhimurium

**DOI:** 10.1038/ncomms12748

**Published:** 2016-09-07

**Authors:** Julie Joseph, Emmanuelle S. Ametepe, Naveen Haribabu, Gerard Agbayani, Lakshmi Krishnan, Alexandre Blais, Subash Sad

**Affiliations:** 1 Faculty of Medicine, Department of Biochemistry, Microbiology, and Immunology, University of Ottawa, Ottawa, Ontario, Canada K1H8M5; 2 National Research Council of Canada-Human Health Therapeutics, Ottawa, Ontario, Canada K1A0R6

## Abstract

Virulent intracellular pathogens, such as the *Salmonella* species, engage numerous virulence factors to subvert host defence mechanisms to induce a chronic infection that leads to typhoid or exacerbation of other chronic inflammatory conditions. Here we show the role of the forkhead transcription factor FoxO3a during infection of mice with *Salmonella* typhimurium (ST). Although FoxO3a signalling does not affect the development of CD8^+^ T cell responses to ST, FoxO3a has an important protective role, particularly during the chronic stage of infection, by limiting the persistence of oxidative stress. Furthermore, FoxO3a signalling regulates ERK signalling in macrophages, which results in the maintenance of a proinflammatory state. FoxO3a signalling does not affect cell proliferation or cell death. Thus, these results reveal mechanisms by which FoxO3a promotes host survival during infection with chronic, virulent intracellular bacteria.

## Abstract (web summary)

FoxO3a signalling has limited influence over acute bacterial infection. Here the authors show that FoxO3a promotes survival of mice in response to chronic *Salmonella* typhimurium infection by restraining oxidative stress and ERK signalling.

## Graphical abstract

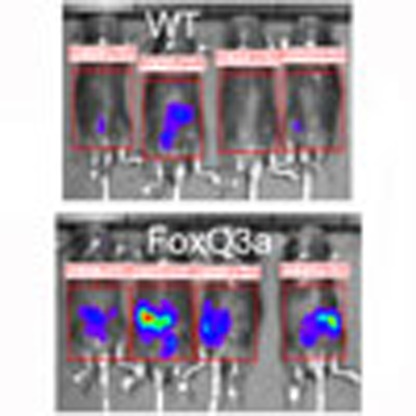

Innate immune cells, including macrophages, neutrophils and monocytes, act as the first line of defence against pathogens^1^. Pattern recognition receptors, such as Toll-like receptors (TLRs), expressed by innate immune cells recognize pathogen associated molecular patterns expressed by a wide range of pathogens^2^. TLR-pathogen associated molecular pattern interactions lead to the activation of nuclear factor-κB (NF-κB)/mitogen-activated protein kinase (MAPK), which results in the expression of inflammatory cytokines that aid in pathogen clearance^3^. Inflammatory responses are subsequently limited by anti-inflammatory cytokines, such as interleukin-10 (IL-10), to prevent excessive tissue damage^4^.

*Salmonella* species are intracellular bacteria that promote typhoid, gastroenteritis, sepsis, inflammatory bowel disease and colon cancer^5^,^6^. *Salmonella* typhimurium (ST) is a gram-negative intracellular bacterium that causes a lethal typhoid-like infection in mice, and takes advantage of the compromised immune system of HIV-1^+^ patients^7^. Approximately 20 million cases of typhoid are reported worldwide annually^8^, and available vaccines have limited efficacy^9^. *Salmonella* reside in the phagosomes of infected cells and create a permissive environment that supports survival of the pathogen^10^,^11^.

Efficient control of ST requires the presence of several host factors including NADPH oxidase, proinflammatory cytokines and activation of adaptive immune cells^12^. ST preferentially infects macrophages and neutrophils^13^,^14^, and replication within macrophages is a mechanism of ST virulence^15^. The production of proinflammatory cytokines (interferon-g (IFN-γ), IL-12 and tumour-necrosis factor (TNF)) and reactive oxygen species (ROS) by macrophages and neutrophils contributes to bacterial killing^16^,^17^,^18^ and their depletion compromises host survival^19^,^20^.

The FoxO family of transcription factors modulates various cellular processes including cell-death and anti-oxidant defence^21^. FoxO3a regulates multiple genes involved in cell-death and cell proliferation^22^. FoxO3a has been shown to negatively regulate CD8^+^ T cell responses in viral infections^23^,^24^. We previously reported that FoxO3a does not promote host survival against the intracellular bacterium, *Listeria monocytogenes* that induces an acute infection, although FoxO3a signalling reduces the CD8^+^ T cell responses to *Listeria monocytogenes*^25^. We consider the possibility that FoxO3a signalling is more important during chronic infections that perturb various host cellular functions more profoundly. We therefore test the role of FoxO3a signalling during infection of mice with virulent ST that causes a chronic infection. Our results indicate that FoxO3a plays a protective role especially in the chronic stage of the infection, by limiting oxidative stress. FoxO3a signalling, in addition, promotes the termination of extracellular signal-regulated kinase (ERK) signalling to induce inflammatory immune responses that are necessary to control infection with virulent intracellular pathogens such as ST.

## Results

### FoxO3a deficiency enhances susceptibility to ST

**Figure 1:**
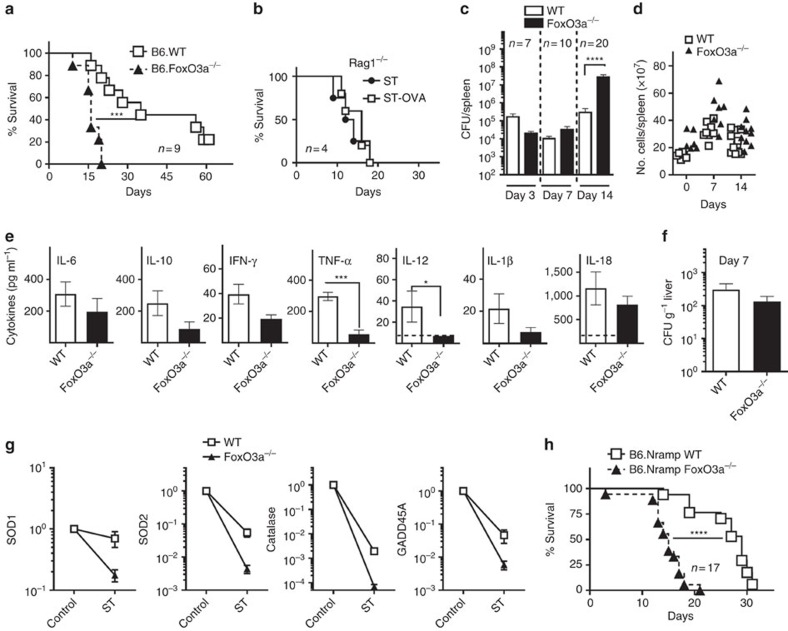
Deficiency of FoxO3a increases susceptibility to ST. (**a**) Survival of WT and FoxO3a^−/−^ C57BL/6 J mice following infection with ST-OVA (10^4^, 9 mice per group). (**b**) Survival of Rag1^−/−^ mice infected (iv) with ST and ST-OVA (4 mice per group). (**c**) Bacterial burden in the spleens of WT and FoxO3a^−/−^ mice at various time intervals post-infection. Results are pooled from 2 to 5 independent experiments with a minimum of 2–3 mice per group. (**d**) Total number of cells/spleen of WT and FoxO3a^−/−^ mice at various time intervals post-infection. Results are pooled from at least three independent experiments with a minimum of 2–3 mice per group. (**e**) Serum cytokine levels in WT and FoxO3a^−/−^ mice at day 7 post-infection with 10^4^ ST-OVA (3 mice per group). (**f**) Bacterial burden in the livers of WT and FoxO3a^−/−^ mice at day 7 post-infection (4 mice per group). (**g**) Modulation of anti-oxidant defence/DNA repair genes in the livers of WT and FoxO3a^−/−^ mice at day 7 post-infection with 10^4^ ST-OVA. Results are pooled from 3 mice (uninfected) or 7–8 mice (+ST, day 7) per group. (**h**) Survival of B6.Nramp WT and B6.Nramp FoxO3a^−/−^ mice following infection with ST (10^3^). Data is pooled from two independent experiments, each with 7–10 mice per group. All graphs depict mean±s.e.m. Survival data were analysed using Mantel–Cox test. All other data was analysed using two-tailed Student's *t*-test (*P< 0.05, ***P<0.001 and ****P<0.0001).

FoxO3a has been shown to influence CD4^+^ and CD8^+^ T cell responses in various infection models^23^,^24^,^25^. We wished to evaluate the impact of FoxO3a during infection with a chronic bacterium that resides in the phagosomes of infected cells. We used a recombinant of ST that resides in the phagosomes and expresses ovalbumin (OVA) (ST-OVA) rapidly into the cytosol of infected cells to evaluate innate as well as acquired immune responses^26^. On infection of either wild type (WT) or FoxO3a^−/−^ C57BL/6 mice with ST-OVA, FoxO3a^−/−^ mice displayed accelerated susceptibility with a median survival of 16 days as opposed to 32 days in case of WT mice (Fig. 1a). Rag-1^−/−^ mice infected with ST or ST-OVA (10^3^, iv) succumbed to the infection suggesting that T cell response is required for protection against ST (Fig. 1b). FoxO3a^−/−^ mice had a comparable bacterial burden to that of WT mice at day 7 post-infection. At day 14 post-infection, the bacterial burden in FoxO3a^−/−^ mice was significantly higher than WT mice (Fig. 1c). FoxO3a^−/−^ spleens were slightly larger than their WT counterparts both before and after infection (Fig. 1d). Thus, our results indicate that when ST infection proceeds for a longer duration, the critical protective role of FoxO3a becomes apparent.

Proinflammatory cytokines play a crucial role in the protection against intracellular pathogens, including ST^16^,^17^,^18^. We analysed serum cytokine levels at day 7, a time point when the bacterial burden was comparable between WT and FoxO3a^−/−^ mice. Interestingly, the levels of critical inflamatory cytokines, TNF and IL-12 were reduced in FoxO3a^−/−^ mice (Fig. 1e). Serum levels of several chemokines including monokine induced by gamma interferon (MIG) and monocyte chemoattractant protein-1 (MCP-1) were also lower in infected FoxO3a^−/−^ mice (Supplementary Fig. 1a). In the liver, FoxO3a^−/−^ mice had significantly reduced expression of various cytokines such as IL-1β, IL-12, TNF and IFN-γ at day 7 post-infection as measured by qRT-PCR; though the expression of SOCS-1 and SOCS-3 were not altered (Supplementary Fig. 1b,c).

At day 7 post-infection, bacterial burden in the livers of FoxO3a^−/−^ mice was comparable to that of WT mice (Fig. 1f). FoxO3a has been shown to promote the transcription of anti-oxidant defence/DNA repair genes in various cell types^27^,^28^. Infection with ST resulted in substantial reduction in the expression of SOD-1, SOD-2, catalase and GADD45A (Fig. 1g), perhaps to allow ROS levels to build-up to control the pathogen. This reduction in the expression of ROS detoxifying genes was exacerbated in the absence of FoxO3a, indicating that FoxO3a promotes the expression of these proteins (Fig. 1g).

The increased susceptibility of C57BL/6 mice to ST has been attributed, at least partially to a mutation in the iron transporter, *Nramp1* gene at position 169 (Gly to Asp)^29^. We therefore used B6 mice carrying a functional Nramp gene (B6.Nramp)^30^, and infected them with ST. Interestingly, B6.Nramp FoxO3a^−/−^ mice succumbed to infection much earlier with a median survival of 15 days in comparison to 28 days in case of B6.Nramp WT mice (Fig. 1h). This data confirms that FoxO3a signalling does have an important, protective role during chronic infection with virulent ST.

### Haematopoietic FoxO3a signalling promotes host protection

**Figure 2:**
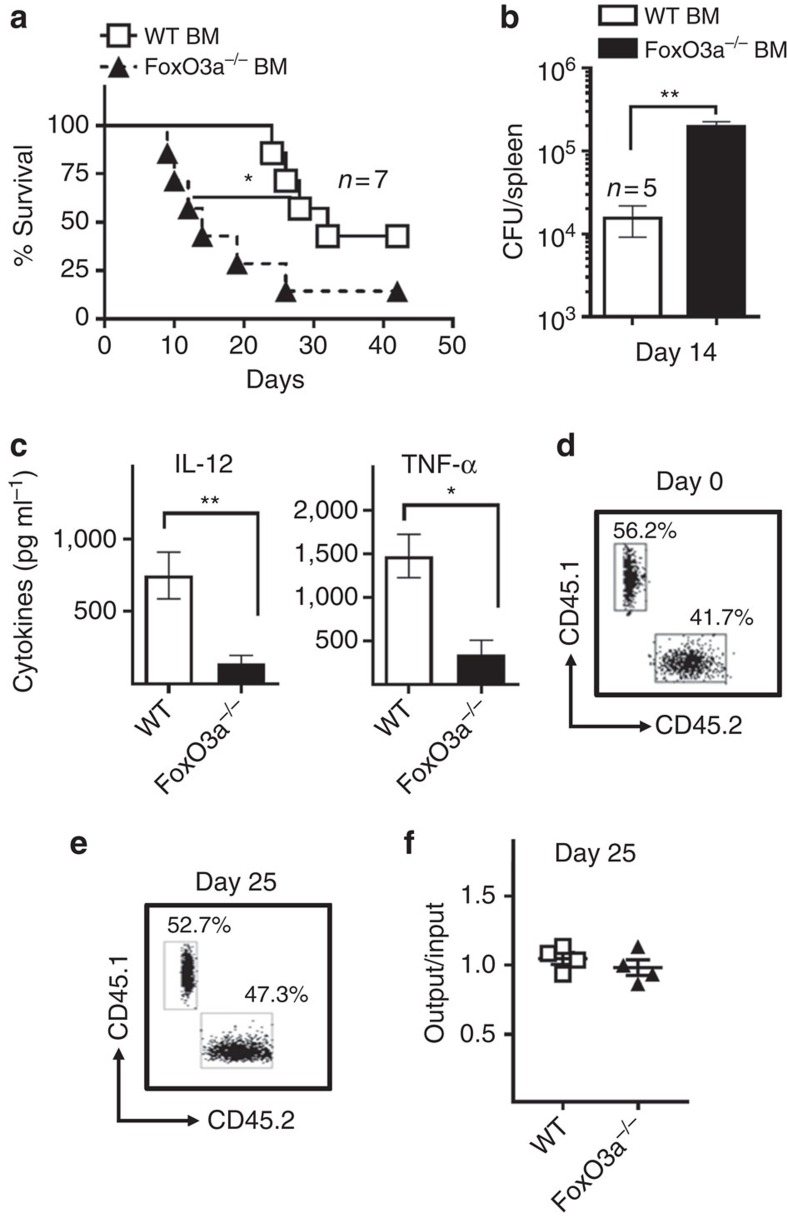
**Susceptibility of FoxO3a**^**−/−**^ **mice is associated with the haematopoietic compartment.** (**a**) Survival of B6 recipient mice reconstituted with WT or FoxO3a^−/−^ bone marrow cells and infected (iv) with 10^4^ ST-OVA (7 mice per group). (**b**) ST burden in the spleens of B6 bone marrow chimera mice reconstituted with WT or FoxO3a^−/−^ bone marrow cells at day 14 post-infection with ST-OVA (5 mice per group). (**c**) Serum cytokine levels in B6 bone marrow chimera mice reconstituted with WT or FoxO3a^−/−^ bone marrow cells (as in Fig. 2b) at day 14 post-infection with ST-OVA (5 mice per group). (**d**) To measure the efficiency of transplantation of WT (CD45.1^+^) and FoxO3a^−/−^ (CD45.2^+^) cells, bone marrow cells was isolated and mixed 1:1 and injected into irradiated WT recipients. Representative FACS plot shows the relative proportions of WT (CD45.1^+^) and FoxO3a^−/−^ (CD45.2^+^) bone marrow cells in the cell mixture before injection. (**e**) Representative FACS plot showing the relative proportions of WT B6.SJL and B6.FoxO3a^−/−^ bone marrow cells in the peripheral blood of irradiated B6 recipient mice at day 25 post-transfer. (**f**) Frequencies of WT B6.SJL and B6.FoxO3a^−/−^ bone marrow cells in the peripheral blood of irradiate B6 recipient mice, normalized to input frequencies (4 mice per group). All graphs depict mean±s.e.m. Survival data analysed using Mantel–Cox test. All other data analysed using two-tailed Student's *t*-test (*P< 0.05, **P<0.01).

We transferred WT or FoxO3a^−/−^ bone marrow cells into irradiated B6 recipient mice to determine whether protection by FoxO3a is mediated through cells in the hematopoietic compartment. Transfer of FoxO3a^−/−^ bone marrow cells into WT recipient mice resulted in accelerated death following infection (Fig. 2a). Median survival of infected FoxO3a^−/−^ bone marrow recipients was 14 days in comparison to 32 days in case of WT bone marrow recipients. Splenic ST burden in FoxO3a^−/−^ bone marrow recipients was higher than WT bone marrow recipients at day 14 post-infection (Fig. 2b). We also observed that irradiated recipients that had received FoxO3a^−/−^ bone marrow cells (Fig. 2b) had much lower levels of IL-12 and TNF in the serum at day 14 post-infection (Fig. 2c). To ensure that the increased susceptibility is not due to defective transplantation of FoxO3a^−/−^ bone marrow cells, we transplanted a mixture of equal numbers of WT (CD45.1^+^CD45.2^−^) and FoxO3a^−/−^ (CD45.1^−^CD45.2^+^) bone marrow cells into irradiated B6 recipient mice (Fig. 2d). Flow cytometry analyses showed that FoxO3a signalling did not impair the transplantation or maintenance of transferred cells (Fig. 2e,f). Taken together, these results indicate that FoxO3a signalling in the haematopoietic compartment promotes the production of proinflammatory cytokines and subsequent control of ST infection in mice.

### FoxO3a does not impact adaptive immune responses to ST

**Figure 3:**
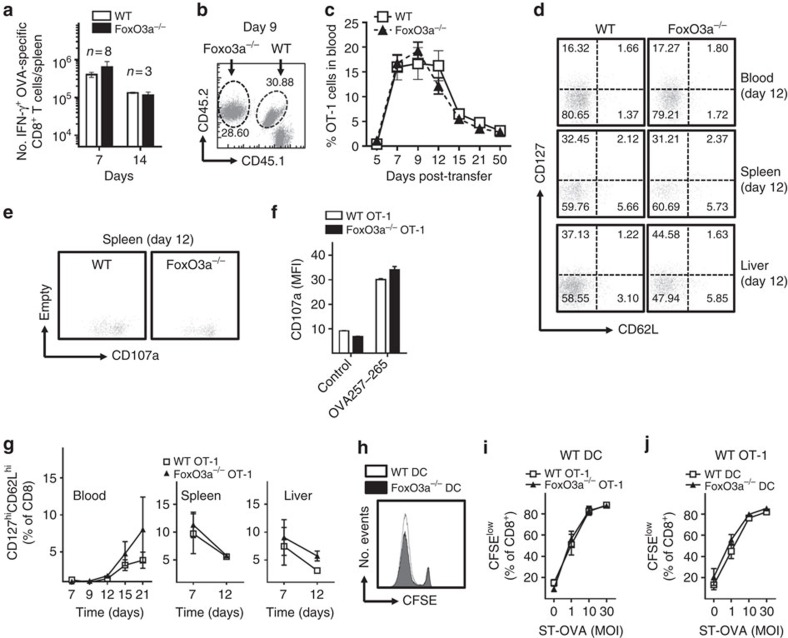
Absence of FoxO3a does not have an impact on adaptive immune responses to ST. (**a**) Number of OVA-specific IFN-γ-secreting CD8^+^ T cells in WT and FoxO3a^−/−^ mice at days 7 and 14 post-infection with ST-OVA (10^4^). Results are pooled from two experiments with a minimum of 3 mice per group. (**b**) Representative FACS plot showing adoptively transferred WT (CD45.1^+^CD45.2^+^) and FoxO3a^−/−^ cells (CD45.1^−^CD45.2^+^) in CD45.1^+^ recipient mice at day 9 post-infection. (**c**) Numbers of WT and FoxO3a^−/−^ OVA-specific CD8^+^ T cells tracked in the blood at different time points post-infection (iv) with ST-OVA (10^4^). Results are pooled from three independent experiments with a minimum of 3 mice per group. (**d**) Representative FACS plots showing similar activation (CD127^low^CD62L^low^) of adoptively transferred WT and FoxO3a^−/−^ CD8^+^ cells at day 12 post-infection. (**e**) Representative FACS plot showing CD107a expression on adoptively transferred WT and FoxO3a^−/−^ cells at day 12 post-infection, following stimulation with OVA-peptide. (**f**) CD107a expression in adoptively transferred WT and FoxO3a^−/−^ OT-1 cells at day 12 post-infection, in the presence or absence of OVA-peptide (2 mice per group) (**g**) Percentages of WT and FoxO3a^−/−^ CD127^hi^CD62L^hi^ CD8^+^ cells in the blood, spleens and livers of recipient mice, at various time intervals following infection with ST-OVA. Liver and spleen data is pooled from 2 mice/time point. Blood data is pooled from a minimum of 3 mice/time point. (**h**) Representative histogram showing CFSE dilution of OT-1 cells in the presence of ST-OVA infected WT/ FoxO3a^−/−^ dendritic cells (DCs). (**i**) Percentage of CFSE^low^ WT and FoxO3a^−/−^ OT-1 cells after culture with WT DCs infected with ST-OVA (various MOI) at 72 h. (**j**) Percentage of CFSE^low^ WT OT-1 cells after culture with ST-OVA infected WT and FoxO3a^−/−^ DCs. Data are pooled from three independent experiments. All graphs depict mean±s.e.m. All data were analysed using two-tailed Student's *t*-test.

Since FoxO3a signalling regulates CD8^+^ T cell response during infection with another intracellular bacterium, *Listeria monocytogenes* without impacting host survival^25^, we considered the possibility that the impairment in the survival of FoxO3a^−/−^ mice following infection with ST may be related to CD8^+^ T cell response. We enumerated IFN-γ producing OVA-specific CD8^+^ cells in WT and FoxO3a^−/−^ mice at various time points after infection, by ELISPOT assay. At both days 7 and 14 post-infection, WT and FoxO3a^−/−^ mice had comparable numbers of IFN-γ producing OVA-specific CD8^+^ cells in the spleen (Fig. 3a). We used an adoptive transfer model to further track and validate the functionality of FoxO3a^−/−^ CD8^+^ T cells during ST infection in a competitive environment (Fig. 3b). The percentages of WT and FoxO3a^−/−^ CD8^+^ T cells following infection were comparable over the course of the study, with no difference observed either in the expansion or in the contraction phase (Fig. 3c). Absence of FoxO3a had no impact on the reduction in the expression of CD62L or CD127 following activation (Fig. 3d,g). Adoptively transferred WT and FoxO3a^−/−^ CD8^+^ T cells showed comparable expression of CD107a (a degranulation marker, which provides a functional readout of the cytolytic potential of CD8^+^ cells) indicating that FoxO3a deficiency did not alter the functionality of OVA-specific CD8^+^ T cells (Fig. 3e,f). *In vitro* antigen presentation experiments indicated that the absence of FoxO3a on DCs or CD8^+^ T cells had no impact on CD8^+^ T cell response during infection with ST (Fig. 3h–j). Taken together, the results indicate that FoxO3a deficiency does not impair CD8^+^ T cell responses to ST.

Comparable numbers of CD4^+^ and CD8^+^ T cells were observed in the livers and peripheral blood of infected WT and FoxO3a^−/−^ mice (Supplementary Fig. 2a,b). However, significantly lower numbers of myeloid cells were observed in the livers and blood of infected FoxO3a^−/−^ mice, indicating that absence of FoxO3a might be associated with defective innate immune responses to ST (Supplementary Fig. 2a,b).

### FoxO3a promotes expression of proinflammatory cytokines

**Figure 4:**
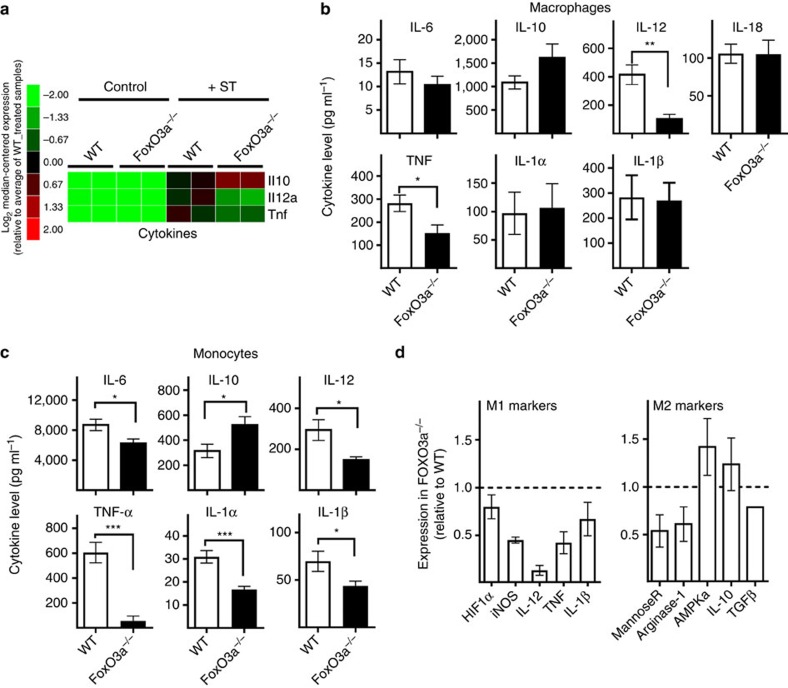
**FoxO3a**^**−/−**^ **macrophages show reduced expression of proinflammatory cytokines.** (**a**) Microarray data depicting the expression of cytokines that showed a change in expression of at least 50% in ST-infected FoxO3a^−/−^ macrophages as compared to ST-infected WT macrophages (10 MOI; 2 mice per treatment). (**b**) Cytokine levels in supernatants of WT and FoxO3a^−/−^ macrophages infected with ST-OVA (10 MOI) at 18–24 h post-infection. Results are pooled from 5 to 7 biological replicates with each being an average of 2–3 experimental replicates. (**c**) Cytokine levels in supernatants of WT and FoxO3a^−/−^ monocytes infected with ST-OVA (10 MOI) at 18–24 h post-infection. Results are pooled from 2 to 4 biological replicates with each being an average of 2–3 experimental replicates. (**d**) Gene expression levels in FoxO3a^−/−^ macrophages relative to WT following infection with ST (10 MOI). Results are pooled from 2 to 4 mice per group. All graphs depict mean±s.e.m. All data were analysed using two-tailed Student's *t*-test (*P<0.05, **P<0.01, ***P<0.001).

Innate immune cells including macrophages have been shown to be critical in controlling ST (ref. 19). Our *in vivo* results suggested that FoxO3a might be required for the generation of optimal innate inflammatory responses, and to determine if this was the case, we tested cytokine production by macrophages and monocytes *in vitro* after infection with ST or ST-OVA (10 multiplicity of infection (MOI)). FoxO3a^−/−^ macrophages produced significantly lower levels of proinflammatory cytokines, IL-12 and TNF, and higher levels of IL-10 following infection (Fig. 4a,b, respectively). Similar results were obtained with FoxO3a^−/−^ monocytes (Fig. 4c) and neutrophils (Supplementary Fig. 3b). ST-OVA at 10 MOI did not result in macrophage cell death and hence we could exclude the possibility that the reduced cytokine levels were due to differential cell death. Despite showing a cytokine profile characteristic of M2 macrophages, gene expression analyses revealed that FoxO3a^−/−^ macrophages had reduced expression of M1 and M2 markers including iNOS, mannose receptor and arginase-1, indicating that FoxO3a^−/−^ macrophages are neither classical M1 nor classical M2 (Fig. 4d). FoxO3a^−/−^ macrophages secreted higher levels of IL-10 and reduced levels of IL-12 and TNF following infection with ST that does not express OVA (Supplementary Fig. 3a), indicating that the impaired proinflammatory response observed in the absence of FoxO3a was independent of OVA expression and secretion. Additionally, FoxO3a motif analyses showed that IL-10, IL-12b and TNF have binding sites for FoxO3a on their proximal promoters (Supplementary Fig. 3c). Taken together, these results indicate that the absence of FoxO3a leads to defective proinflammatory cytokine production by myeloid cells in response to ST.

### FoxO3a does not impact glycolytic switch in response to ST

**Figure 5:**
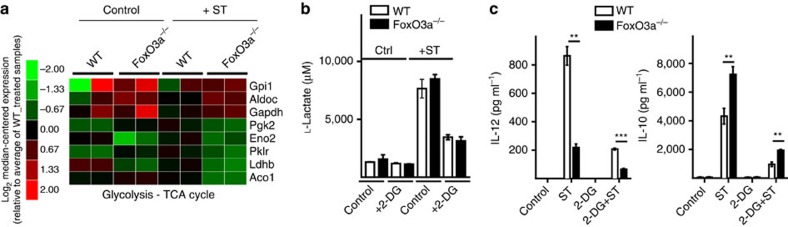
Absence of FoxO3a does not impact the glycolytic switch in response to ST. (**a**) Microarray data depicting the expression of glycolysis-TCA cycle genes that showed a change in expression of at least 50% in ST-infected FoxO3a^−/−^ macrophages as compared with ST-infected WT macrophages (10 MOI; 2 mice per treatment). (**b**) L-lactate levels in WT and FoxO3a^−/−^ macrophages infected with ST (10 MOI) in the presence or absence of 1 mM 2-DG. Data are representative of two independent experiments. (**c**) Expression of IL-12 and IL-10 in WT and FoxO3a^−/−^ macrophages infected with ST (10 MOI) in the presence or absence of 1 mM 2-DG. Data are pooled from two independent experiments. All graphs depict mean±s.e.m. All data were analysed using two-tailed Student's *t*-test (**P<0.01, ***P<0.001).

Lipopolysaccharide (LPS) treatment of macrophages has been shown to be associated with a metabolic switch to glycolysis (Warburg effect) and increased glycolysis has been reported to contribute to cytokine production^31^. Analysis of gene expression by DNA microarrays indicated that the absence of FoxO3a led to a differential expression of several genes involved in glycolysis–TCA cycle (Fig. 5a). Since absence of FoxO3a has been reported to impair glycolysis^32^, we measured glycolysis in FoxO3a^−/−^ macrophages in the presence of ST. Measurement of L-lactate (end product of glycolysis) levels indicated that infection with ST results in enhancement in glycolysis, and that the absence of FoxO3a does not have any impact (Fig. 5b). Addition of 2-DG, an inhibitor of glycolysis, reduced the levels of L-lactate (Fig. 5b), and resulted in a drastic reduction in the expression of cytokines (Fig. 5c). Even when glycolysis was inhibited, FoxO3a^−/−^ macrophages still secreted decreased levels of IL-12 and increased levels of IL-10 suggesting the impaired cytokine production in FoxO3a^−/−^ cells is independent of glycolysis (Fig. 5c and Supplementary Fig. 4).

### FoxO3a does not impact p65/AKT/p38/JNK activation

**Figure 6:**
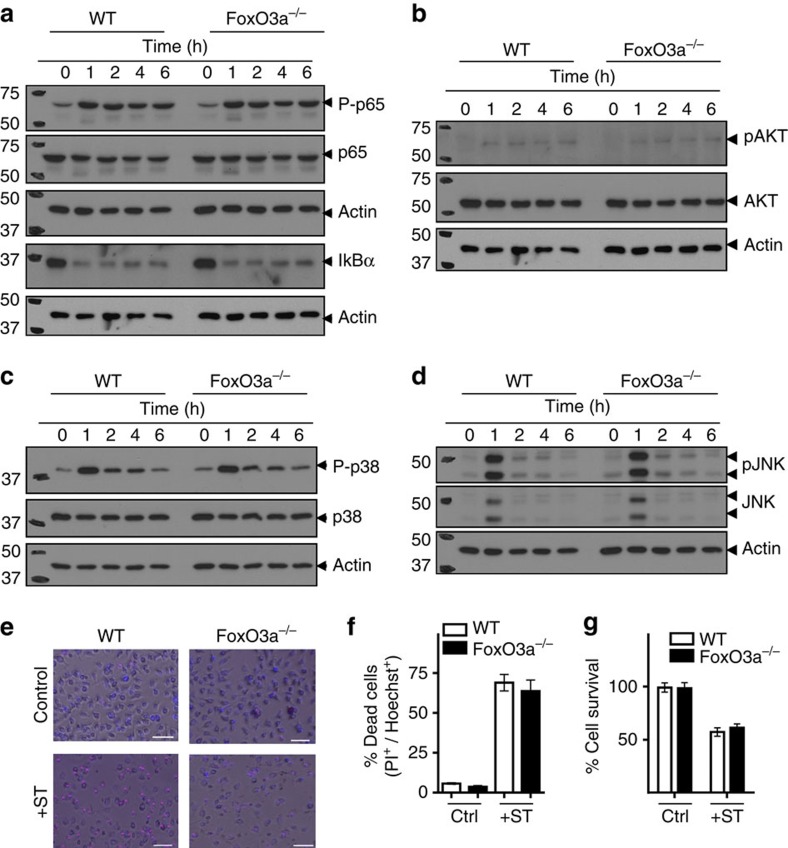
FoxO3a deficiency does not impact p65/AKT/p38/JNK activation. (**a**) Western blots of macrophages at various time intervals post-infection with ST-OVA (10 MOI) for the expression of phospho-p65,p65 and IκBα. Western blots for phospho-AKT,AKT (**b**); phospho-p38, p38 (**c**); and phospho-JNK, JNK (**d**). WT and FoxO3a^−/−^ macrophages were infected with ST-OVA (10 MOI) and lysed for western blotting at various time intervals post-infection (**a**–**d**). All western blotting data is representative of two independent experiments. (**e**) Cell-death in WT and FoxO3a^−/−^ macrophages infected with ST (100 MOI) at 6 h post-infection as measured by PI/Hoechst staining. Scale bars, 50 μm. (**f**) Quantification of data from **e**. (**g**) Survival of WT and FoxO3a^−/−^ macrophages infected with ST-OVA (100 MOI) at 18–24 h post-infection as measured by neutral red uptake. Data are pooled from three biological replicates, each being an average of 2–3 experimental replicates. Graphs depict mean±s.e.m. All data were analysed using two-tailed Student's *t*-test.

Expression of proinflammatory cytokines occurs mainly through the activation of NF-κB pathway. Alternate pathways including PI3K-AKT and MAPK (consisting of p38, JNK and ERK) also contribute to inflammatory cytokine production^33^,^34^. We evaluated the activation of NF-κB/AKT/MAPK pathways by western blotting to determine if proinflammatory cytokine production in response to ST was regulated by FoxO3a via modulation of one of these pathways. The kinetics of p65 phosphorylation and concurrent IκBα degradation were identical between WT and FoxO3a^−/−^ macrophages (Fig. 6a and Supplementary Figs 5 and 8a,b), suggesting that NF-κB signalling is similar in WT and FoxO3a^−/−^ macrophages. Similar phosphorylation of AKT, p38 and JNK was observed in WT and FoxO3a^−/−^ macrophages following infection with ST (Fig. 6b–d and Supplementary Fig. 8c–e). The data collectively indicate that impaired expression of proinflammatory cytokines in FoxO3a^−/−^ macrophages following infection with ST is not related to NF-κB, AKT, p38 or JNK signalling pathways. Since cell-death following infection has been considered as a key mechanism of virulence of ST (ref. 35), we measured cell-death of macrophages after infection with higher MOI of ST (100 MOI) which induced significant cell death. Our results indicate that WT as well as FoxO3a^−/−^ macrophages undergo similar extent of cell-death (Fig. 6e–g).

### FoxO3a regulates ERK signalling in macrophages

**Figure 7:**
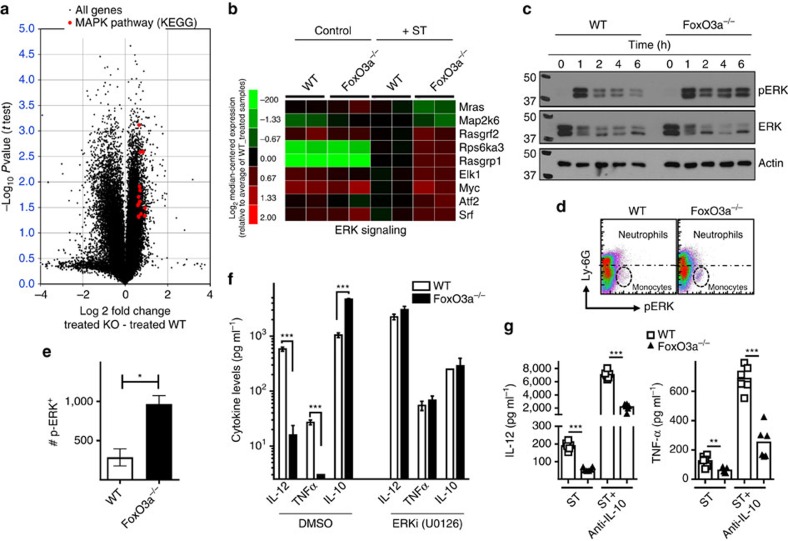
FoxO3a regulates ERK signalling in macrophages. (**a**) Volcano plot showing the fold change in treated KO-WT on the x-axis, and the *P* value associated to this change on the y-axis. Red dots represent 14 MAPK genes (from the Kyoto Encyclopedia of Genes and Genomes) that are up regulated at least 50% with a *P* value of 0.05 or better. (**b**) Microarray data depicting the expression of ERK signalling genes that showed a change in expression of at least 50% in ST-infected FoxO3a^−/−^ macrophages as compared to ST-infected WT macrophages (10 MOI; 2 mice per treatment). (**c**) Western blots for phospho-ERK and ERK. WT and FoxO3a^−/−^ macrophages were infected with ST-OVA (10 MOI) and lysed for western blotting at various time intervals post-infection. Data are representative of two independent experiments. (**d**) Representative dot-plot showing phospho-ERK expression in WT and FoxO3a^−/−^ bone marrow cells at day 7 post-infection (gated on CD11b^+^ cells). (**e**) Bar graph showing the number of phospho-ERK^+^LyG^-^ cells/10^5^ bone marrow cells (three mice per group). (**f**) Cytokine levels as measured in the supernatants of WT and FoxO3a^−/−^ macrophages infected with ST-OVA (10 MOI) in the absence/presence of ERK inhibitor U-0126 at 18-24 h post-infection. Data are representative of at least three biological replicates with each being an average of 2–3 technical replicates. (**g**) IL-12 and TNF expression in the supernatants of infected WT and FoxO3a^−/−^ macrophages treated with anti-IL-10 antibody (10 μg ml^−1^), at 18–24 h post-treatment. Results are pooled from two independent experiments. All graphs depict mean±s.e.m. All data were analysed using two-tailed Student's *t*-test (*P< 0.05, **P<0.01, ***P<0.001).

Analysis of gene expression by DNA microarrays indicated that on infection with ST, a number of MAPK pathway genes are up-regulated in FoxO3a^−/−^ macrophages in comparison to WT macrophages; with 14 genes (from the Kyoto Encyclopedia of Genes and Genomes) up-regulated by 50% and with a *P* value of 0.003 (*t*-test, two-tailed unpaired; Fig. 7a). Several genes upstream of Ras-ERK signalling including Rasgrp1 and Rasgrf2, were expressed at higher levels in infected FoxO3a^−/−^ macrophages, indicating that FoxO3a regulates the expression of these genes upon infection (Fig. 7b). FoxO3a motif analyses showed that both Rasgrp1 and Rasgrf2 have FoxO3a sequence elements within their proximal promoters, suggesting that they might be direct targets of FoxO3a (Supplementary Fig. 6a). Expression of several transcription factors downstream of ERK signalling including Elk1, Myc, ATF-2 and Srf were also significantly higher in infected FoxO3a^−/−^ macrophages, pointing to an enhancement of ERK signalling in the absence of FoxO3a (Fig. 7b). Phosphorylation of ERK was induced in infected WT macrophages and declined significantly at later time intervals. In contrast, FoxO3a^−/−^ macrophages displayed sustained activation of ERK signalling (Fig. 7c and Supplementary Figs 6b and 8f). We isolated bone marrow cells from WT and FoxO3a^−/−^ mice at day 7 post-infection and analysed ERK phosphorylation using phospho-flow cytometry. Infected FoxO3a^−/−^ mice had higher numbers of p-ERK^+^ CD11b^+^ Ly6G^−^ cells, indicating that the absence of FoxO3a leads to enhanced ERK signalling *in vivo* (Fig. 7d,e).

We next investigated whether the reduced expression of proinflammatory cytokines in FoxO3a^−/−^ macrophages was due to sustained ERK signalling. Inhibition of ERK signalling with the commonly used ERK inhibitor U-0126 restored the expression of inflammatory cytokines in FoxO3a^−/−^ macrophages to levels similar to WT macrophages (Fig. 7f). Similar results were obtained with a second ERK inhibitor, PD0325901 (Supplementary Fig. 6c). Western blots showed a near total inhibition of ERK phosphorylation by both inhibitors (Supplementary Figs 6d and 8g). These results indicate that the enhanced/sustained activation of ERK due to the absence of FoxO3a signalling leads to reduced expression of proinflammatory cytokines. Our results are in line with previous studies that have reported ERK activation to negatively regulate proinflammatory cytokine production^36^,^37^.

ERK inhibition led to a reduction in IL-10 production, and IL-10 has been shown to directly inhibit the expression of inflammatory cytokines^38^. To investigate whether the reduced expression of proinflammatory cytokines in FoxO3a^−/−^ macrophages was mediated by higher expression of IL-10, we neutralized the expression of IL-10 using an antibody and evaluated the impact on the expression of IL-12 and TNF expression. Inhibition of IL-10 resulted in an increase in the expression of IL-12 and TNF by both WT and FoxO3a-deficient cells (Fig. 7g). Despite leading to a significant reduction in IL-10 levels (Supplementary Fig. 6e), anti- IL-10 treatment could not restore the expression of IL-12 and TNF levels in FoxO3a^−/−^ macrophages to WT levels (Fig. 7g), indicating that the rescue observed with ERK inhibition is not through IL-10.

Aberrant activation of ERK in FoxO3a^−/−^ neural stem cells has been attributed to decreased expression of negative regulators of Ras-ERK signalling^32^. ERK signalling has been shown to be negatively regulated by dual specificity phosphatases (DUSPs), diacylglycerol kinases (DGKs) and proteins such as Sprouty/Spred^39^,^40^,^41^. We evaluated the messenger RNA (mRNA) levels of these proteins in infected macrophages to determine if FoxO3a regulated their expression. FoxO3a^−/−^ cells, in contrast to WT cells, expressed significantly lower levels of DGK-ζ and Spry-2 (Supplementary Fig. 6f). Magnitude of reduction of DGK-ζ mRNA was significantly higher in FoxO3a^−/−^ macrophages over the course of the infection (Supplementary Fig. 6g). FoxO3a motif analyses showed that both DGK-ζ and Spry-2 have binding sites for FoxO3a on their proximal promoters (Supplementary Fig. 6a), suggesting that FoxO3a could positively regulate the expression of these genes via direct DNA binding. Taken together, our results indicate that in the absence of FoxO3a, deregulation of multiple genes in the ERK signalling pathway (Rasgrf2, Rasgrp1, DGK-ζ and Spry-2) leads to increased signalling; resulting in impaired inflammatory cytokine production by ST infected macrophages.

### Oxidative stress in FoxO3a^−/−^ mice promotes susceptibility

**Figure 8:**
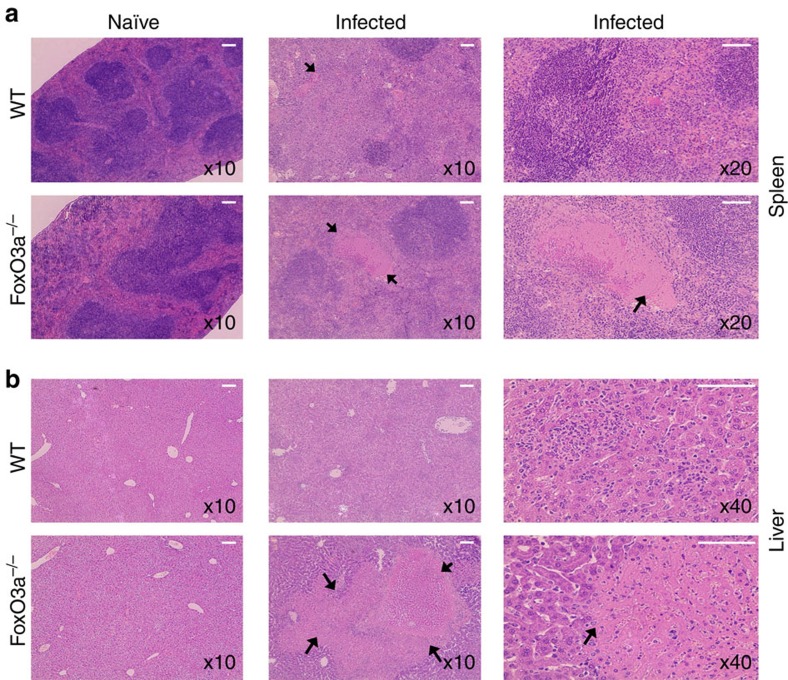
Absence of FoxO3a signalling leads to increased tissue damage and host pathology. (**a**) Representative images (haematoxylin and eosin) of spleen-sections of naïve and infected (day 14, ST-OVA) WT and FoxO3a^−/−^ mice. Arrows indicate splenic lesions, which were larger and more frequent in FoxO3a^−/−^ mice. Data are representative of three mice per group. (**b**) Representative images (haematoxylin and eosin) of liver-sections of naïve and infected (day 14, ST-OVA) WT and FoxO3a^−/−^ mice. Data are representative of three mice per group. Arrows indicate areas of necrosis which were numerous and larger in livers of FoxO3a^−/−^ mice. Scale bar, 50 μm.

We harvested the spleens (Fig. 8a) and livers (Fig. 8b) of infected mice on day 14 post-infection and performed haematoxylin and eosin staining to evaluate tissue pathology. Naïve WT and FoxO3a^−/−^ mice showed normal tissue architecture of spleen and liver. Infected FoxO3a^−/−^ mice had more necrotic lesions in the spleen and liver, leading to large areas of tissue loss (Fig. 8a,b).

**Figure 9:**
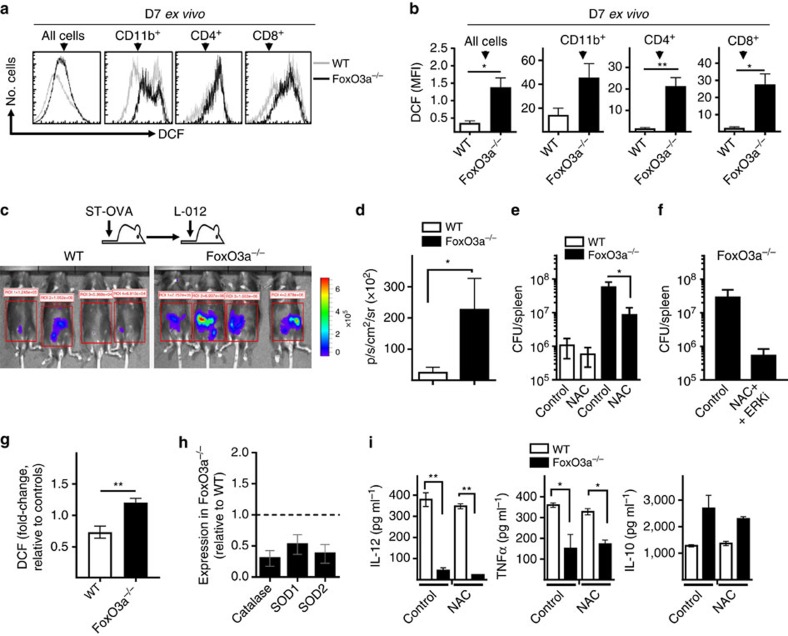
**Oxidative stress in FoxO3a**^**−/−**^ **mice contributes to disease susceptibility.** (**a**,**b**) ROS levels as measured by H_2_DCFDA staining of infected spleen cells from WT and FoxO3a^−/−^ mice at day 7 post-infection. Representative histograms (**a**) and bar graphs quantifying the data are shown (**b**). MFI data is pooled from three mice per group. (**c**,**d**) At day 7 post-infection with ST-OVA, WT and FoxO3a^−/−^ mice were injected with L-012 to measure reactive species levels *in vivo* by bioluminescence imaging (BLI). Data are representative of two independent experiments, each with 2–4 mice per group. (**e**) Day14 bacterial burden in the spleens of WT and FoxO3a^−/−^ mice treated with 40 mM NAC over the course of infection. Results are pooled from two independent experiments with a total of 7–11 mice per group. (**f**) Bacterial burden in the spleens of FoxO3a^−/−^ mice left untreated or treated with NAC and ERKi over the course of infection (a minimum of 4 mice per group). (**g**) Fold-induction of ROS as measured by DCF staining in WT and FoxO3a^−/−^ macrophages following infection with ST-OVA (10 MOI). Data are pooled from three independent experiments. (**h**) Expression of ROS detoxifying genes in FoxO3a^−/−^ macrophages relative to WT. Results are pooled from 4 mice per group. (**i**) Cytokine levels in the supernatants of WT and FoxO3a^−/−^ macrophages infected with ST-OVA (10 MOI) in the presence or absence of 200 μM NAC. Data are representative of two biological replicates, each being an average of 2–3 experimental replicates. All graphs depict mean±s.e.m. All data were analysed using two-tailed Student's *t*-test (*P<0.05, **P<0.01).

Infection with ST has been shown to be associated with increased oxidative stress^13^,^42^ and increased ROS levels have been implicated in the development of liver diseases/pathology^43^. The reduction of SOD-1, SOD-2, catalase and GADD45A was much more pronounced in the livers of FoxO3a^−/−^ mice following infection with ST (Fig. 1g). Decreased expression of anti-oxidant genes in infected FoxO3a^−/−^ mice led us to question if these mice harbour higher ROS levels during *Salmonella* infection. FoxO3a^−/−^ splenocytes (day 7 post-infection) showed increased levels of ROS as measured by DCF staining. Increase in ROS levels was observed in FoxO3a^−/−^ CD8^+^ T cells, CD4^+^ T cells and myeloid cells (Fig. 9a,b). We tested the reactive species levels *in vivo* by bioluminescence imaging using L-012, a luminol based chemiluminescent probe. Since differences in bacterial burden can affect ROS measurements, we measured ROS levels at day 7 post-infection, when the bacterial burden in the spleen and liver was comparable between WT and FoxO3a^−/−^ mice (Fig. 1c,f). Enhanced luminescence levels were observed in infected FoxO3a^−/−^ mice compared with WT mice (Fig. 9c,d). N-acetyl cysteine (NAC) treatment in mice led to a partial reduction in ROS levels *in vivo* (Supplementary Fig. 7a,b). Inhibition of ROS *in vivo* using NAC caused a reduction in bacterial burden in FoxO3a^−/−^ mice (Fig. 9e), which was reduced even further by ERK inhibition (Fig. 9f and Supplementary Fig. 7c). Taken together, these data suggest that FoxO3a^−/−^ mice harbour increased levels of reactive species, leading to a higher degree of oxidative stress during ST infection, which impairs bacterial control.

Infected FoxO3a-deficient macrophages (Fig. 9g) and neutrophils (Supplementary Fig. 7d) expressed higher levels of ROS (as measured by DCF staining). Furthermore, FoxO3a^−/−^ macrophages expressed reduced levels of the anti-oxidant enzymes SOD-1, SOD-2 and Catalase (Fig. 9h), similar to what was observed *in vivo*. Analysis of gene expression by DNA microarrays in ST infected macrophages revealed a significant reduction in the expression of several genes of the glutathione detoxification system in FoxO3a^−/−^ macrophages (Supplementary Fig. 7e), suggesting a major breakdown of ROS detoxification systems in the absence of FoxO3a. Furthermore, FoxO3a motif analyses showed that catalase, SOD1 and SOD2 have binding sites for FoxO3a on their proximal promoters (Supplementary Fig. 7f), suggesting that FoxO3a might positively regulate the expression of these genes via direct DNA binding. Increased ROS levels have been shown to induce ERK phosphorylation^44^,^45^. To determine if the increased ROS levels in FoxO3a^−/−^ cells could contribute to prolonged ERK signalling and reduced proinflammatory cytokine production, we infected macrophages with ST in the presence or absence of NAC. Inhibition of ROS with NAC could not rescue poor proinflammatory cytokine production in FoxO3a^−/−^ macrophages (Fig. 9i). Thus our results indicate that increased ROS levels and impaired proinflammatory cytokine production are mutually exclusive and that together, they severely impair the host's ability to clear ST infection.

## Discussion

Pathogens express numerous virulence mechanisms to subvert the army of various types of immune cells; however, the immune system efficiently controls a myriad of pathogens^46^,^47^. FoxO3a, a key transcription factor that promotes expression of various genes involved in cell signalling, was shown to previously have only a modest impact on acquired immune response in various models^23^,^24^,^25^. We hypothesized that FoxO3a signalling might become paramount particularly during chronic infection that puts extensive stress on the immune system due to persistent engagement of various cell signalling mechanisms. We have therefore tested the role of FoxO3a signalling during infection with ST, a chronic, virulent intracellular bacterium. Our results indicate that FoxO3a-signalling enables better bacterial control by limiting oxidative stress. It also regulates ERK signalling in macrophages to induce the expression of high levels of inflammatory cytokines. Proinflammatory cytokine responses are imperative for clearance of intracellular pathogens^16^,^17^,^18^, and any modulation of this pathway is bound to have significant impact on host survival.

Increased ROS levels have been reported to promote bacterial killing in the early stages of *Salmonella* infection^48^,^49^ and the increased levels of ROS in FoxO3a-deficient mice appeared to promote better control initially. S*almonella* detoxifying enzymes have been described to successfully protect against host oxidative burst, and *Salmonella* are also able to escape to more permissive environments^13^,^50^. Despite bacterial burden being similar to WT mice at day 7 post-infection, FoxO3a^−/−^ mice had high ROS levels (Fig. 9c), which correlated with reduced expression of ROS-scavenging enzymes, indicative of defective ROS detoxification. During *Porphyromonas gingivalis* infection, host oxidative response was reported to increase morbidity and mortality, by increasing systemic inflammation^51^. The fact that NAC treatment of FoxO3a^−/−^ mice resulted in a decrease of bacterial burden indicates that ROS might not contribute to bacterial clearance at later stages of the infection. Instead, persistently high levels of ROS could lead to excessive host pathology and reduced host survival.

FoxO1 and FoxO3a are the main FoxO transcription factors expressed in the immune system. As opposed to FoxO1 which is expressed highly in lymphoid cells, FoxO3a is expressed at high levels in myeloid cells^52^,^53^ suggesting that FoxO3a deficiency might have a more pronounced impact in the myeloid compartment. FoxO3a signalling regulates CD8^+^ T cell responses in various models^23^,^24^,^25^,^54^. We failed to observe any impact of FoxO3a in CD8^+^ T cell response to ST. Although T cells play an important role in promoting clearance of ST in WT hosts, this occurs after the first 3 weeks of infection^55^,^56^. Since FoxO3a^−/−^ mice were moribund early on during infection, this suggests that a modulation of innate immune response was the reason for increased susceptibility.

FoxO proteins have been reported to regulate the expression of inflammatory markers in multiple models; however the literature has been contradictory. FoxO1 has been shown to positively regulate inflammation in murine macrophages through modulation of TLR4 signalling^57^. A recent study by Seiler *et al*. reported that FoxO3 is expressed in respiratory epithelium in response to bacterial infections and that knockdown of FoxO transcription factors impairs the release of innate immune factors by respiratory epithelial cells^58^. The caveat is that these studies have been done either in the context of FoxO1 deficiency or have used combined inhibition of FoxO1/FoxO3a, making it hard to delineate the individual contributions of the two proteins. A study that specifically looked at FoxO3a deficiency in the context of cigarette smoke induced inflammation observed that absence of FoxO3a led to down-regulation of antioxidant genes and to exaggerated inflammatory responses^59^. Our results show for the first time that independent of FoxO1, FoxO3a plays a crucial role in regulating immune responses to an intracellular bacterium, by promoting the expression of proinflammatory cytokines in innate immune cells. Our data indicate that FoxO3a plays an indispensable role in host survival against ST by regulating ROS levels and enhancing the expression of inflammatory cytokines.

FoxO3a deficiency resulted in lower levels of cytokines/chemokines and reduced numbers of myeloid cells in the liver and blood, which is indicative of a defect in the generation/egress of myeloid cells in/from the bone marrow following infection with ST. IFN-γ promotes infection-induced myelopoiesis in the context of intracellular bacterial infection^60^ and FoxO3a^−/−^ mice had lower levels of serum IFN-γ, MIG and MCP-1. Both MIG and MCP-1 have been shown to be important in controlling bacterial infections, with MCP-1-deficient mice succumbing earlier to *Salmonella enterica* infection^61^. The reduced chemokine levels could lead to altered chemotaxis and account for the reduced numbers of circulating myeloid cells observed in infected FoxO3a^−/−^ mice. A recent study reported that bacterial infection causes cell-death of kupffer cells and that their replacement by blood monocyte derived macrophages contribute to antibacterial immunity while restoring tissue integrity^62^. Thus reduced myeloid cell numbers in FoxO3a^−/−^ mice could severely impair the replacement of liver resident macrophages, leading to decreased bacterial clearance and exacerbated tissue damage.

Serum/tissue levels of IL-10 were slightly reduced in ST infected FoxO3a^−/−^ mice; however, the reduction was not as severe as that observed for proinflammatory cytokines such as TNF and IL-12. Furthermore, the reduced numbers/defective migration of myeloid cells in FoxO3a^−/−^ mice following ST infection may drive reduction in cytokine levels overall. These technical complexities were mitigated by infecting macrophages *in vitro* and equalizing the cell numbers, which decisively revealed increased IL-10 expression by FoxO3a^−/−^ myeloid cells.

Altered cytokine levels are usually associated with modulation of NF-κB signalling, and FoxO3a was reported to negatively regulate NF-κB signalling in T cells, although the mechanism was not clear^63^. Similar results were obtained in a recent study where absence of FoxO3a in tumour associated dendritic cells led to enhanced NF-κB nuclear translocation and subsequent expression of inflammatory cytokines including IL-12 (ref. 64). In contrast, we observed reduced IL-12 expression in FoxO3a^−/−^ macrophages and failed to notice any modulation of NF-κB signalling. It is possible that the differences are related to the different cell-types (T cells and tumour associated dendritic cells versus macrophages) and the different experimental set-ups (autoimmunity and tumour versus bacterial infection). Reduction in the expression of proinflammatory cytokines in FoxO3a^−/−^ macrophages could be rescued by the inhibition of ERK signalling; suggesting that over-activation of the ERK signalling was responsible for the phenotype of FoxO3a^−/−^ macrophages, in the context of ST infection. Several downstream targets of ERK signalling pathway, including MSK1/2, c-Fos and CREB have been reported to promote anti-inflammatory phenotype^36^,^37^,^65^.

Aberrant activation of ERK has been reported in FoxO3a-deficient neural stem cells and has been attributed to decreased expression of negative regulators of Ras-ERK signalling^32^. Rasgrp1 and Rasgrf2, which positively regulate Ras-ERK signalling, were up-regulated in ST infected FoxO3a^−/−^ macrophages, indicating that it is not just the negative regulators, the expression of which is impacted in the absence of FoxO3a. Multiple proteins (Sprouty/Spred, DUSPs and DGKs) act in concert to inactivate ERK signalling^39^,^40^,^41^. DGK-ζ appears to be the major isoform expressed in macrophages^66^. We failed to observe any modulation in the expression of DUSP5/DUSP6 in infected FoxO3a^−/−^ cells, but we cannot rule out the possibility that other DUSPs and/or protein phosphatases might be involved. Following infection, FoxO3a^−/−^ cells had significantly lower levels of DGK-ζ mRNA. It is conceivable that FoxO3a regulates DGK-ζ by increasing the stability of its mRNA. DGKζ-deficient mice show reduced expression of IL-12/TNF and increased susceptibility to *Toxoplasma gondii*, lending further support to our hypothesis that FoxO3a promotes proinflammatory cytokine production at least partially through its modulation of DGKζ (ref. 66).

Most of the genes deregulated in infected FoxO3a^−/−^ macrophages have predicted binding sites for FoxO3a on their proximal promoters. Whether FoxO3a regulates the expression of these genes via direct binding to the promoters requires further investigation. FoxO3a has been reported to bind to the promoter sequence of SOD-2, thereby activating the promoter. A model was proposed wherein FoxO3a induces the expression of PGC-1α and co-operates with this protein to bind to the promoter sequences of target genes resulting in maximal expression of anti-oxidant enzymes^67^. We have shown that IL-10, IL-12b and TNF have predicted FoxO3a binding sites on their proximal promoters suggesting that FoxO3a binding could play a role in the transcription of these genes. Recently it was reported that Nrf-2 inhibited the expression of inflammatory cytokines (IL6, IL12b and IL-1β, not TNF), in response to LPS (ref. 68) by binding to the promoters of target genes. We did not see any modulation of Nrf-2 in infected FoxO3a^−/−^ macrophages.

Modulation of the inflammatory phenotype of macrophages by FoxO3a signalling did not have any impact on their susceptibility to cell-death or IL-1β/ΙL-18 production, which indicates that activation of inflammasomes was not impacted by FoxO3a. Pyroptosis has been shown to be a key mechanism of cell-death of macrophages following infection with ST, which leads to inflammation induced by IL-1β/ΙL−18 processing^69^,^70^. Our results thus indicate that not all pathways of inflammation are impacted by FoxO3a.

The immune system mounts a swift inflammatory response to control rapidly proliferating, virulent pathogens. *Salmonella* species are intracellular bacteria that reside in the phagosomes of infected cells and promote typhoid, gastroenteritis, sepsis, inflammatory bowel disease and colon cancer^5^,^6^. Development of a swift and efficient proinflammatory immune response is necessary for controlling such pathogens^16^,^17^,^18^. Our study reveals that by reducing oxidative stress and by modulating ERK signalling, FoxO3a transcription factor tilts the balance towards increased inflammatory responses to control intracellular bacteria.

## Methods

### Bacterial strains

ST expressing OVA in phagosome was generated by incorporating pKKOVA plasmid into ST, whereas for cytosolic antigenic delivery OVA along with its fusion protein, YopE and chaperone, SycE was incorporated into ST using pHR-OVA plasmid^26^. Colony-forming units were calculated by performing serial dilutions on brain heart infusion (BHI) plates.

### Mice and infections

All animal procedures were performed on approval by the University of Ottawa Animal Care and Veterinary Committee. Age and gender matched mice were used at 6–10 weeks of age. C57BL/6 J and B6.SJL mice were obtained from Jackson Laboratory (Bar Harbor, Maine, USA). Generation of FoxO3a^−/−^ mice has been described before^25^. B6.Nramp mice were provided by Dr Greg Barton (University of California, Berkeley)^30^. B6.Nramp FoxO3a^−/−^ mice were generated by crossing B6.Nramp mice with FoxO3a^−/−^ mice. For immunizations, frozen stocks were thawed and diluted in 0.9% saline, and mice were inoculated iv.

### Bacterial burden

Whole spleens from infected mice were homogenized using frosted glass slides. livers (0.5 g) were weighed out and homogenized in 1 ml PBS using Cell MagNA Lyser (Roche, Switzerland). Colony-forming units were then determined by plating 100 μl aliquots of 10-fold serial dilutions on BHI-Agar plates.

### Bone marrow chimera

10^7^ bone marrow cells from WT or FoxO3a^−/−^ mice were injected into irradiated B6 recipient mice, which were then maintained on antibiotics and special rodent chow diet for a period of 3 weeks. Recipient mice were then provided with regular chow and maintained for 2 months to ensure proper bone marrow repopulation. Mice were then infected with 10^4^ ST-OVA iv.

### Generation of bone marrow derived macrophages

Bone marrow cells were obtained by flushing the tibia and femurs of mice. Cells were plated in RPMI-1640 containing 8% foetal bovine serum (FBS) and differentiated into macrophages by the addition of 5 ng ml^−1^ M-CSF (R&D, MN, USA).

### Infection of cells

Cells were infected with ST-OVA or ST. Lower multiplicity of infection (10 MOI) was used for measurement of cytokines. Higher MOI (100 MOI) was used when measuring cell death. Cells were centrifuged for 7 min to enhance bacterial uptake. Infection usually lasted 30 min. Gentamicin (Life technologies, CA, USA) containing media was added and left overnight to eliminate extracellular bacteria. Supernatants were collected at 18–24 h post-infection and frozen at −80 °C. For NAC/ U-0126/ PD0325901 treatments, cells were treated with NAC (200 μM; Sigma-Aldrich, MO, USA), U-0126 (40 μM; Cell Signaling Technology, MA, USA), PD0325901 (0.2 μM; Selleckchem, USA) 1 h before infection. For 2-DG treatment, cells were treated with 2-DG (1 mM; Sigma- Aldrich, MO, USA) 3 h before infection. For anti-IL-10 treatment, cells were treated with anti-IL-10 (10 μg ml^−1^; BD) concurrently with ST. Cells were maintained in media containing inhibitors/antibody till 18–24 h post-infection, when supernatants were collected.

### Measurement of transplant efficiency and cell survival

Bone marrow cells from B6.SJL (CD45.1^+^CD45.2^−^) and FoxO3a^−/−^ (CD45.1^−^CD45.2^+^) mice were counted and mixed 1:1. The mixed cell suspension was then injected into irradiated B6 recipient mice (10^7^ cells per mouse). Flow Cytometry was performed on the injection mix to confirm the relative proportions of CD45.1^+^ and CD45.2^+^ cells, at the start of the experiment. Recipient mice were maintained on antibiotics and special rodent chow diet for a period of 3 weeks, after which they were provided with regular chow for the rest of the duration of the experiment. Transplant efficiency was measured by determining the frequencies of CD45.1^+^ (WT) and CD45.2^+^ (FoxO3a^−/−^) cell populations in the peripheral blood, at day 25 post-transfer and comparing it to the input frequencies.

### Adoptive transfer for evaluation of CD8^+^ T cell response

CD45.1^+^ CD45.2^+^ OT-1 mice were bred in-house. FoxO3a^−/−^ OT-1 mice were generated by mating OT-1 (CD45.2^+^) mice with FoxO3a^−/−^ mice. Spleen cells were obtained from WT OT-1 (CD45.1^+^CD45.2^+^) and FoxO3a^−/−^ OT-1 (CD45.1^−^CD45.2^+^) mice, mixed 1:1 and then injected (iv, 5 × 10^4^ cells per mouse) into B6.SJL (CD45.1^+^CD45.2^−^) recipient mice. The following day, recipient mice were infected with 10^4^ ST-OVA, and OVA-specific CD8^+^ T cells were then tracked in blood using antibodies against CD45.1 and CD45.2 at different time points post- infection. Expression of CD127 and CD62L on donor T cells was analysed by flow cytometry.

### CD107a degranulation assay

Spleen cells were resuspended in RPMI-1640 supplemented with 8% FBS and 50 IU ml^−1^ IL-2. They were then stimulated with OVA peptide (257–264) (1 μg ml^−1^) for 4 h at 37 °C in the presence of GolgiStop (BD) and anti-CD107a antibody (eBioscience). Cells were then stained with anti-CD45.1, anti-CD45.2, anti-CD8 Abs and the expression of CD107a evaluated by flow cytometry.

### *In vitro* antigen presentation assay

DCs were isolated from naïve spleens using a PE-CD11c-positive selection kit from Stemcell Technologies (BC, Canada). 5 × 10^4^ DCs were seeded on a 96-well plate in RPMI-1640 supplemented with 8% FBS and then infected with ST-OVA at MOIs 1, 10 and 30 for 30 min, followed by 2 h treatment with gentamicin (50 μg ml^−1^). Carboxyfluorescein succinimidyl ester (CFSE)-labelled OT-1 CD8^+^ T cells (5 × 10^4^) were purified using a CD8 negative selection kit (Stemcell Technologies) and incubated with the infected DCs for a period of 72 h. Reduction in CFSE expression on CD8^+^ T cells was evaluated by flow cytometry (CyAn ADP, Beckman Coulter, CA, USA).

### Flow cytometry

Spleens were homogenized using frosted glass slides. Livers were homogenized using a filter and plunger and the lymphocytes were isolated via Ficoll separation. Blood samples were subjected to red blood cell lysis. In all cases, cells were suspended at 10^6^/tube and were blocked with anti-CD16/32 at 4 °C. After 10 min, cells were stained with the appropriate antibody cocktail for 30 min. Antibodies were obtained from eBioscience. Cells were washed with PBS and fixed in 2.0% paraformaldehyde and acquired on fluorescence-activated cell sorting (FACS) Canto (BD) or alternatively on CyAn ADP (Beckman Coulter, CA, USA). Data was analysed using FlowJo (TreeStar, OR, USA) or Kaluza (Beckman Coulter) software.

### Phospho-flow cytometry

Spleen and bone marrow cells were blocked with blocking solution (PBS with 10% goat serum and 20% FBS) containing anti-CD16/32 for 10 min at 4 °C and then stained with surface antibodies against CD11b and Ly6G for 30 min. Cells were then fixed and permeabilized using the Cytofix/Cytoperm Fixation/ Permeabilization solution kit (BD) and methanol and stained with PE Mouse Anti-ERK1/2 (pT202/pY204) for 30 min at room temperature (BD). Cells were then acquired on a BD LSRFortessa.

### Isolation of monocytes and neutrophils from bone marrow

Monocytes were isolated from bone marrow using EasySep Mouse Monocyte Enrichment Kit (Stemcell Technologies). Neutrophils were isolated using Ficoll separation.

### Reactive species measurements

For *in vitro* measurements, neutrophils/macrophages were seeded on 96-well plates at a concentration of 10^5^ cells per well and infected with 10 MOI of ST-OVA. At indicated time points following infection, cells were loaded with 20 μM H_2_DCFDA (Molecular Probes) in serum-free RPMI-1640 and incubated for 30 min at 37 °C. Cells were then washed twice with PBS, suspended in fresh RPMI-1640, no phenol red and readings were acquired at an excitation wavelength of 495 nm using a FilterMax F5 plate reader (Molecular Devices, CA, USA).

For *ex vivo* measurements, spleens were obtained from infected mice and single-cell suspensions were prepared. spleen cells (10^6^) were then loaded with 20 μM H_2_DCFDA (Molecular Probes) in serum-free RPMI-1640 and incubated for 30 min at 37 °C. Cells were then suspended in fresh RPMI-1640, no phenol red and acquired immediately on CyAn ADP (Beckman Coulter). For *in vivo* ROS measurements, ST-OVA infected mice were anaesthetized with isoflurane, and injected (ip, 20 μg g^−1^ of body weight) with L-012 (Wako, Osaka, Japan) which was dissolved in PBS. Bioluminescence signal was measured at 6 min post-injection of L-012 using an IVIS Spectrum imaging system (Perkin Elmer, MA, USA).

### *In vivo* treatments

For NAC treatment, mice were randomized to *ad lib* drinking water supplemented with 40 mM NAC or regular drinking water. Treatment was started 1 day before ST-OVA infection and continued until day 14 post-infection, when the mice were killed. Drinking water was replenished every 3–4 days. For ERKi treatment *in vivo*, mice received 100 μl of ERKi (PD0325901) at a concentration of 10 mg kg^−1^ of body weight. ERKi (PD0325901) was prepared fresh daily, in a solution of 0.5% hydroxypropyl methylcellulose plus 0.2% Tween 80. Mice that received regular drinking water were given 100 μl of a solution of 0.5% hydroxypropyl methylcellulose plus 0.2% Tween 80. Treatment was commenced 2 days before infection with ST-OVA and was given daily by oral gavage.

### Western blotting

Cells were lysed in 1% SDS lysing buffer containing 1% β-ME and boiled immediately after for 10 min. Western blot analyses were performed using standard protocol. Antibodies used were as follows: phospho-p65 (Cell Signaling #3033), p65 (Cell Signaling #8242), IκB (Cell Signaling #4814), phospho-AKT (Cell Signaling #9275), AKT (Cell Signaling #9272), phospho-p38 (Cell Signaling #4511), p38 (Cell Signaling #8690), phospho-JNK (Cell Signaling #4668), JNK (Cell Signaling #9258), phospho-ERK (Cell Signaling #4370), ERK (Cell Signaling #4695) and β-actin (Santa Cruz Biotechnology #sc-81178).

### Histopathology

Spleens and livers were harvested and fixed in neutral buffered 10% formalin, embedded in paraffin and processed for routine histopathological examination. Vertical sections (5 μm thick) were stained with haematoxylin and eosin. Images were acquired on a Zeiss Axio Imager.M2 microscope.

### Blood collection

Blood was collected via saphenous/sub-mandibular bleeding or via cardiac puncture. For Flow Cytometric analyses, blood was collected in anticoagulant tubes (BD microtainer with Lithium-Heparin; BD). For serum cytokine measurements, blood was collected in serum separator tubes (BD microtainer with serum separator; BD) and the serum was separated by centrifugation and frozen at −80 ^o^C.

### PI/Hoechst staining

Bone marrow derived macrophages were plated in 96-well plates at a concentration of 10^5^ cells per well on day 6. Cells were then infected and at desired time points, stained with Hoechst (2.5 μg ml^−1^; Life Technologies) and propidium iodide (1:10 dilution; BD) and incubated at 37 °C for 15–20 min. Immunofluorescence images were acquired on a Zeiss Axio Observer.D1 microscope. Cell counting was performed using Infinity Analyse software (Lumenera Corporation, ON, Canada).

### Neutral red uptake assay

A 10% solution of Neutral Red (Sigma- Aldrich) was prepared in RPMI-1640 containing 8% FBS. The solution was filtered to minimize the formation of crystals and was then added to macrophages. The cells were incubated with the solution for up to 2 h at 37 °C. After removal of the stain solution, the cells were washed with PBS and the dye extracted/ solubilized using a solution of ethanol and acetic acid. The absorbance of the solubilized dye was then measured at 570 nm using a FilterMax F5 plate reader (Molecular Devices).

### Glycolysis cell-based assay

Cells were plated in RPMI-1640 containing 8% FBS. Supernatants were collected from treated/infected cells at 18–24 h post-infection. L-lactate, the end product of glycolysis was measured using the Glycolysis Cell-Based Assay kit from Cayman Chemical (Michigan, USA), according to manufacturer's instructions. Absorbance was then measured at 450 nm using a FilterMax F5 plate reader (Molecular Devices).

### Cytokine/chemokine measurements

Cytokine/chemokine measurements were done using either ELISA (eBioscience, CA, USA) or Cytometric Bead Array (BD). IL-1β, TNF-α, IL-10, IL-6 and IL-12p70 were measured using BD OptEIA kits or BD CBA Flex sets according to manufacturer's instructions. IL-1α was measured using Mouse IL-1α ELISA Max Standard kit (BioLegend, CA, USA) or CBA flex set (BD). Other cytokines and chemokines were measured using CBA (BD). IL-18 was measured on a MAGPIX multiplex reader (Luminex, USA) using ProcartaPlex Mouse IL-18 Simplex kit (eBioscience).

### Gene expression profiling

RNA samples were collected at 6 h post-infection using RNeasy Mini Kit (Qiagen). Transcriptomic analyses were performed essentially as described. Total RNA (200 ng) from the various cell populations was reverse transcribed and fluorescently amplified using the Agilent Low-input Quick Amp single color labeling kit. Labelled cRNA was hybridized to Agilent-028005 SurePrint G3 Mouse GE 8x60K Microarray (GPL10787). Data were analysed using Expander 7, using log-transformation and quantiles normalization between the eight samples. Heat-maps were prepared using Java TreeView with microarray probes representing genes belonging to pathways of interest and using expression data that have been median-subtracted across RNA samples. The raw and normalized data have been deposited in the Gene Expression Omnibus (GEO) data base (accession number: GSE82212).

### Gene promoter analyses

The programme oPOSSUM-3 was used to identify phylogenetically conserved FoxO3 binding sites within the regulatory regions of genes of interest. Hits to the JASPAR position-weight matrix MA0157 for FoxO3 were searched for. Only hits within the first 2,800 base pairs of upstream promoter regions and with a matrix score threshold of at least 85% were retained.

### Real-time PCR

Organs were harvested from mice and immediately frozen in RNAlater stabilization reagent (Qiagen, Limburg, Netherlands). RNA was prepared using RNeasy Mini Kit (Qiagen) according to manufacturer's instructions. Alternatively, cells were lysed in TRIzol reagent (Life Technologies) and RNA was prepared according to manufacturer's instructions. RNA concentrations were determined using a FilterMax F5 plate reader (Molecular Devices). complementary DNA was prepared using SuperScript III Reverse Transcriptase (Life Technologies) following TURBO DNase treatment (Life Technologies) of the RNA, according to manufacturer's instructions. Wherever possible, exon–exon spanning primers were used to avoid amplification of genomic DNA. Real-time PCR was done using SYBR Green PCR Master Mix (Life Technologies) and the reactions were run on an ABI 7500 PCR machine. Primer sequences are shown in Supplementary Table 1.

### Statistics

Data were analysed in GraphPad Prism (GraphPad Software, California, USA). Survival curves were analysed using Mantel–Cox test; all other data were analysed by two-tailed unpaired Student's *t* tests. All graphs represent mean±s.e.m. *P* values<0.05 were considered significant.

### Data availability

Microarray data that support the findings of this study have been deposited in GEO with the primary accession code GSE82212. The authors declare that all other data supporting the findings of this study are available within the article and its Supplementary information files.

## Additional information

**How to cite this article:** Joseph, J. *et al*. Inhibition of ROS and upregulation of inflammatory cytokines by FoxO3a promotes survival against *Salmonella* typhimurium. *Nat. Commun.* 7:12748 doi: 10.1038/ncomms12748 (2016).

## Supplementary Material

Supplementary Information Supplementary Figures 1-8 and Supplementary Table 1.

## References

[b1] Dempsey P. W., Vaidya S. A. & Cheng G. The art of war: innate and adaptive immune responses. Cell. Mol. Life sci. 60, 2604–2621 (2003). 14685686 10.1007/s00018-003-3180-y PMC11138847

[b2] Kumar H., Kawai T. & Akira S. Pathogen recognition by the innate immune system. Int. Rev. Immunol. 30, 16–34 (2011). 21235323 10.3109/08830185.2010.529976

[b3] West A. P., Koblansky A. A. & Ghosh S. Recognition and signaling by toll-like receptors. Annu. Rev. Cell Dev. Biol. 22, 409–437 (2006). 16822173 10.1146/annurev.cellbio.21.122303.115827

[b4] Cyktor J. C. & Turner J. Interleukin-10 and immunity against prokaryotic and eukaryotic intracellular pathogens. Infect. Immun. 79, 2964–2973 (2011). 21576331 10.1128/IAI.00047-11 PMC3147550

[b5] Caygill C. P., Hill M. J., Braddick M. & Sharp J. C. Cancer mortality in chronic typhoid and paratyphoid carriers. Lancet 343, 83–84 (1994). 7903779 10.1016/s0140-6736(94)90816-8

[b6] Coburn B., Grassl G. A. & Finlay B. B. Salmonella, the host and disease: a brief review. Immunol. Cell Biol. 85, 112–118 (2007). 17146467 10.1038/sj.icb.7100007

[b7] Gordon M. A. . Invasive non-typhoid salmonellae establish systemic intracellular infection in HIV-infected adults: an emerging disease pathogenesis. Clin. Infect. Dis. 50, 953–962 (2010). 20180702 10.1086/651080

[b8] Crump J. A., Luby S. P. & Mintz E. D. The global burden of typhoid fever. Bull. World Health Organ. 82, 346–353 (2004). 15298225 PMC2622843

[b9] Guzman C. A. . Vaccines against typhoid fever. Vaccine 24, 3804–3811 (2006). 16278037 10.1016/j.vaccine.2005.07.111

[b10] Knodler L. A. & Steele-Mortimer O. Taking possession: biogenesis of the Salmonella-containing vacuole. Traffic 4, 587–599 (2003). 12911813 10.1034/j.1600-0854.2003.00118.x

[b11] Bakowski M. A., Braun V. & Brumell J. H. Salmonella-containing vacuoles: directing traffic and nesting to grow. Traffic 9, 2022–2031 (2008). 18778407 10.1111/j.1600-0854.2008.00827.x

[b12] Valdez Y., Ferreira R. B. & Finlay B. B. Molecular mechanisms of Salmonella virulence and host resistance. Curr. Top. Microbiol. Immunol. 337, 93–127 (2009). 19812981 10.1007/978-3-642-01846-6_4

[b13] Aussel L. . Salmonella detoxifying enzymes are sufficient to cope with the host oxidative burst. Mol. Microbiol. 80, 628–640 (2011). 21362067 10.1111/j.1365-2958.2011.07611.x

[b14] Geddes K., Cruz F. & Heffron F. Analysis of cells targeted by Salmonella type III secretion in vivo. PLoS Pathog. 3, e196 (2007). 18159943 10.1371/journal.ppat.0030196 PMC2151088

[b15] Fields P. I., Swanson R. V., Haidaris C. G. & Heffron F. Mutants of *Salmonella typhimurium* that cannot survive within the macrophage are avirulent. Proc. Natl Acad. Sci. USA 83, 5189–5193 (1986). 3523484 10.1073/pnas.83.14.5189 PMC323916

[b16] Nauciel C. & Espinasse-Maes F. Role of gamma interferon and tumor necrosis factor alpha in resistance to *Salmonella typhimurium* infection. Infect. Immun. 60, 450–454 (1992). 1730475 10.1128/iai.60.2.450-454.1992 PMC257648

[b17] Jouanguy E. . IL-12 and IFN-gamma in host defense against mycobacteria and salmonella in mice and men. Curr. Opin. Immunol. 11, 346–351 (1999). 10375558 10.1016/s0952-7915(99)80055-7

[b18] Price J. D. . Gamma interferon-independent effects of interleukin-12 on immunity to *Salmonella enterica serovar Typhimurium*. Infect. Immun. 75, 5753–5762 (2007). 17875635 10.1128/IAI.00971-07 PMC2168367

[b19] O'Brien A. D., Scher I. & Formal S. B. Effect of silica on the innate resistance of inbred mice to Salmonella typhimurium infection. Infect. Immun. 25, 513–520 (1979). 226477 10.1128/iai.25.2.513-520.1979 PMC414481

[b20] Vassiloyanakopoulos A. P., Okamoto S. & Fierer J. The crucial role of polymorphonuclear leukocytes in resistance to Salmonella dublin infections in genetically susceptible and resistant mice. Proc. Natl Acad. Sci. USA 95, 7676–7681 (1998). 9636209 10.1073/pnas.95.13.7676 PMC22720

[b21] Eijkelenboom A. & Burgering B. M. FOXOs: signalling integrators for homeostasis maintenance. Nat. Rev. Mol. Cell Biol. 14, 83–97 (2013). 23325358 10.1038/nrm3507

[b22] van der Vos K. E. & Coffer P. J. The extending network of FOXO transcriptional target genes. Antioxid. Redox Signal. 14, 579–592 (2011). 20673124 10.1089/ars.2010.3419

[b23] Sullivan J. A., Kim E. H., Plisch E. H., Peng S. L. & Suresh M. FOXO3 regulates CD8 T cell memory by T cell-intrinsic mechanisms. PLoS. Pathog. 8, e1002533 (2012). 22359505 10.1371/journal.ppat.1002533 PMC3280979

[b24] Dejean A. S. . Transcription factor Foxo3 controls the magnitude of T cell immune responses by modulating the function of dendritic cells. Nat. Immunol. 10, 504–513 (2009). 19363483 10.1038/ni.1729 PMC2712214

[b25] Tzelepis F. . Intrinsic role of FoxO3a in the development of CD8+ T cell memory. J. Immunol. 190, 1066–1075 (2013). 23277488 10.4049/jimmunol.1200639 PMC3815477

[b26] Tzelepis F. . Modulation of antigenic location converts chronic into acute infection by forcing CD8(+) T cell recognition. Cell Rep. 2, 1710–1721 (2012). 23219554 10.1016/j.celrep.2012.10.024

[b27] Marinkovic D. . Foxo3 is required for the regulation of oxidative stress in erythropoiesis. J. Clin. Invest. 117, 2133–2144 (2007). 17671650 10.1172/JCI31807 PMC1934587

[b28] Yalcin S. . Foxo3 is essential for the regulation of ataxia telangiectasia mutated and oxidative stress-mediated homeostasis of hematopoietic stem cells. J. Biol. Chem. 283, 25692–25705 (2008). 18424439 10.1074/jbc.M800517200

[b29] Vidal S. M., Pinner E., Lepage P., Gauthier S. & Gros P. Natural resistance to intracellular infections: Nramp1 encodes a membrane phosphoglycoprotein absent in macrophages from susceptible (Nramp1 D169) mouse strains. J. Immunol. 157, 3559–3568 (1996). 8871656

[b30] Arpaia N. . TLR signaling is required for Salmonella typhimurium virulence. Cell 144, 675–688 (2011). 21376231 10.1016/j.cell.2011.01.031 PMC3063366

[b31] Kelly B. & O'Neill L. A. Metabolic reprogramming in macrophages and dendritic cells in innate immunity. Cell Res. 25, 771–784 (2015). 26045163 10.1038/cr.2015.68 PMC4493277

[b32] Yeo H. . FoxO3 coordinates metabolic pathways to maintain redox balance in neural stem cells. EMBO J. 32, 2589–2602 (2013). 24013118 10.1038/emboj.2013.186 PMC3791369

[b33] Makela S. M., Strengell M., Pietila T. E., Osterlund P. & Julkunen I. Multiple signaling pathways contribute to synergistic TLR ligand-dependent cytokine gene expression in human monocyte-derived macrophages and dendritic cells. J. Leukoc. Biol. 85, 664–672 (2009). 19164128 10.1189/jlb.0808503

[b34] Banerjee A. & Gerondakis S. Coordinating TLR-activated signaling pathways in cells of the immune system. Immunol. Cell Biol. 85, 420–424 (2007). 17637696 10.1038/sj.icb.7100098

[b35] Lindgren S. W., Stojiljkovic I. & Heffron F. Macrophage killing is an essential virulence mechanism of *Salmonella typhimurium*. Proc. Natl Acad. Sci. USA 93, 4197–4201 (1996). 8633040 10.1073/pnas.93.9.4197 PMC39511

[b36] Agrawal S. . Cutting edge: different Toll-like receptor agonists instruct dendritic cells to induce distinct Th responses via differential modulation of extracellular signal-regulated kinase-mitogen-activated protein kinase and c-Fos. J. Immunol. 171, 4984–4989 (2003). 14607893 10.4049/jimmunol.171.10.4984

[b37] Tomczak M. F. . Defective activation of ERK in macrophages lacking the p50/p105 subunit of NF-kappaB is responsible for elevated expression of IL-12 p40 observed after challenge with Helicobacter hepaticus. J. Immunol. 176, 1244–1251 (2006). 16394015 10.4049/jimmunol.176.2.1244

[b38] Rahim S. S., Khan N., Boddupalli C. S., Hasnain S. E. & Mukhopadhyay S. Interleukin-10 (IL-10) mediated suppression of IL-12 production in RAW 264.7 cells also involves c-rel transcription factor. Immunology 114, 313–321 (2005). 15720433 10.1111/j.1365-2567.2005.02107.x PMC1782084

[b39] Caunt C. J. & Keyse S. M. Dual-specificity MAP kinase phosphatases (MKPs): shaping the outcome of MAP kinase signalling. FEBS J. 280, 489–504 (2013). 22812510 10.1111/j.1742-4658.2012.08716.x PMC3594966

[b40] Sakane F., Imai S., Kai M., Yasuda S. & Kanoh H. Diacylglycerol kinases: why so many of them? Biochim. Biophys. Acta 1771, 793–806 (2007). 17512245 10.1016/j.bbalip.2007.04.006

[b41] Kim H. J. & Bar-Sagi D. Modulation of signalling by Sprouty: a developing story. Nat. Rev. Mol. Cell Biol. 5, 441–450 (2004). 15173823 10.1038/nrm1400

[b42] Sem X. & Rhen M. Pathogenicity of *Salmonella enterica* in Caenorhabditis elegans relies on disseminated oxidative stress in the infected host. PLoS ONE 7, e45417 (2012). 23028994 10.1371/journal.pone.0045417 PMC3461013

[b43] Jaeschke H. Reactive oxygen and mechanisms of inflammatory liver injury: Present concepts. J. Gastroenterol. Hepatol. 26, 173–179 (2011). 21199529 10.1111/j.1440-1746.2010.06592.x

[b44] Mehdi M. Z., Azar Z. M. & Srivastava A. K. Role of receptor and nonreceptor protein tyrosine kinases in H2O2-induced PKB and ERK1/2 signaling. Cell Biochem. Biophys. 47, 1–10 (2007). 17406055 10.1385/cbb:47:1:1

[b45] Aikawa R. . Oxidative stress activates extracellular signal-regulated kinases through Src and Ras in cultured cardiac myocytes of neonatal rats. J. Clin. Invest. 100, 1813–1821 (1997). 9312182 10.1172/JCI119709 PMC508367

[b46] Brodsky I. E. & Medzhitov R. Targeting of immune signalling networks by bacterial pathogens. Nat. Cell Biol. 11, 521–526 (2009). 19404331 10.1038/ncb0509-521

[b47] Iwasaki A. & Medzhitov R. Control of adaptive immunity by the innate immune system. Nat. Immunol. 16, 343–353 (2015). 25789684 10.1038/ni.3123 PMC4507498

[b48] Mastroeni P. . Antimicrobial actions of the NADPH phagocyte oxidase and inducible nitric oxide synthase in experimental salmonellosis. II. Effects on microbial proliferation and host survival in vivo. J. Exp. Med. 192, 237–248 (2000). 10899910 10.1084/jem.192.2.237 PMC2193252

[b49] Shiloh M. U. . Phenotype of mice and macrophages deficient in both phagocyte oxidase and inducible nitric oxide synthase. Immunity 10, 29–38 (1999). 10023768 10.1016/s1074-7613(00)80004-7

[b50] Burton N. A. . Disparate impact of oxidative host defenses determines the fate of Salmonella during systemic infection in mice. Cell Host Microbe 15, 72–83 (2014). 24439899 10.1016/j.chom.2013.12.006

[b51] Mydel P. . Roles of the host oxidative immune response and bacterial antioxidant rubrerythrin during *Porphyromonas gingivalis* infection. PLoS Pathog. 2, e76 (2006). 16895445 10.1371/journal.ppat.0020076 PMC1522038

[b52] Dejean A. S., Hedrick S. M. & Kerdiles Y. M. Highly specialized role of Forkhead box O transcription factors in the immune system. Antioxid. Redox Signal. 14, 663–674 (2011). 20673126 10.1089/ars.2010.3414 PMC3021368

[b53] Wu C. . BioGPS: an extensible and customizable portal for querying and organizing gene annotation resources. Genome Biol. 10, R130 (2009). 19919682 10.1186/gb-2009-10-11-r130 PMC3091323

[b54] Van Grevenynghe J. . Transcription factor FOXO3a controls the persistence of memory CD4(+) T cells during HIV infection. Nat. Med. 14, 266–274 (2008). 18311149 10.1038/nm1728

[b55] Luu R. A. . Delayed expansion and contraction of CD8+ T cell response during infection with virulent Salmonella typhimurium. J. Immunol. 177, 1516–1525 (2006). 16849458 10.4049/jimmunol.177.3.1516 PMC4015949

[b56] Srinivasan A., Foley J., Ravindran R. & McSorley S. J. Low-dose *Salmonella* infection evades activation of flagellin-specific CD4 T cells. J. Immunol. 173, 4091–4099 (2004). 15356159 10.4049/jimmunol.173.6.4091

[b57] Fan W. . FoxO1 regulates Tlr4 inflammatory pathway signalling in macrophages. EMBO J. 29, 4223–4236 (2010). 21045807 10.1038/emboj.2010.268 PMC3018786

[b58] Seiler F. . FOXO transcription factors regulate innate immune mechanisms in respiratory epithelial cells. J. Immunol. 190, 1603–1613 (2013). 23315071 10.4049/jimmunol.1200596

[b59] Hwang J. W. . FOXO3 deficiency leads to increased susceptibility to cigarette smoke-induced inflammation, airspace enlargement, and chronic obstructive pulmonary disease. J. Immunol. 187, 987–998 (2011). 21690325 10.4049/jimmunol.1001861 PMC3131437

[b60] MacNamara K. C. . Infection-induced myelopoiesis during intracellular bacterial infection is critically dependent upon IFN-gamma signaling. J. Immunol. 186, 1032–1043 (2011). 21149601 10.4049/jimmunol.1001893 PMC3178067

[b61] Depaolo R. W., Lathan R., Rollins B. J. & Karpus W. J. The chemokine CCL2 is required for control of murine gastric *Salmonella enterica* infection. Infect. Immun. 73, 6514–6522 (2005). 16177325 10.1128/IAI.73.10.6514-6522.2005 PMC1230974

[b62] Bleriot C. . Liver-resident macrophage necroptosis orchestrates type 1 microbicidal inflammation and type-2-mediated tissue repair during bacterial infection. Immunity 42, 145–158 (2015). 25577440 10.1016/j.immuni.2014.12.020

[b63] Lin L., Hron J. D. & Peng S. L. Regulation of NF-kappaB, Th activation, and autoinflammation by the forkhead transcription factor Foxo3a. Immunity 21, 203–213 (2004). 15308101 10.1016/j.immuni.2004.06.016

[b64] Thompson M. G. . FOXO3-NF-kappaB RelA protein complexes reduce proinflammatory cell signaling and function. J. Immunol. 195, 5637–5647 (2015). 26561547 10.4049/jimmunol.1501758 PMC4670825

[b65] Feng G. J. . Extracellular signal-related kinase (ERK) and p38 mitogen-activated protein (MAP) kinases differentially regulate the lipopolysaccharide-mediated induction of inducible nitric oxide synthase and IL-12 in macrophages: Leishmania phosphoglycans subvert macrophage IL-12 production by targeting ERK MAP kinase. J. Immunol. 163, 6403–6412 (1999). 10586030

[b66] Liu C. H. . Diacylglycerol kinase zeta regulates microbial recognition and host resistance to Toxoplasma gondii. J. Exp. Med. 204, 781–792 (2007). 17371930 10.1084/jem.20061856 PMC2118554

[b67] Olmos Y. . Mutual dependence of Foxo3a and PGC-1alpha in the induction of oxidative stress genes. J. Biol. Chem. 284, 14476–14484 (2009). 19324885 10.1074/jbc.M807397200 PMC2682896

[b68] Kobayashi E. H. . Nrf2 suppresses macrophage inflammatory response by blocking proinflammatory cytokine transcription. Nat. Commun. 7, 11624 (2016). 27211851 10.1038/ncomms11624 PMC4879264

[b69] Fink S. L. & Cookson B. T. Pyroptosis and host cell death responses during Salmonella infection. Cell. Microbiol. 9, 2562–2570 (2007). 17714514 10.1111/j.1462-5822.2007.01036.x

[b70] Broz P. & Monack D. M. Molecular mechanisms of inflammasome activation during microbial infections. Immunol. Rev. 243, 174–190 (2011). 21884176 10.1111/j.1600-065X.2011.01041.x PMC3170129

