# Supplementary material for: Inhibition of ROS and upregulation of inflammatory cytokines by FoxO3a promotes survival against Salmonella typhimurium
Source: Nat Commun. 2016 Sep 7;7:12748. doi: 10.1038/ncomms12748 (PMC5023958; doi:10.1038/ncomms12748)
Supplement: Supplementary Information — Supplementary Figures 1-8 and Supplementary Table 1. [file ncomms12748-s1.pdf]

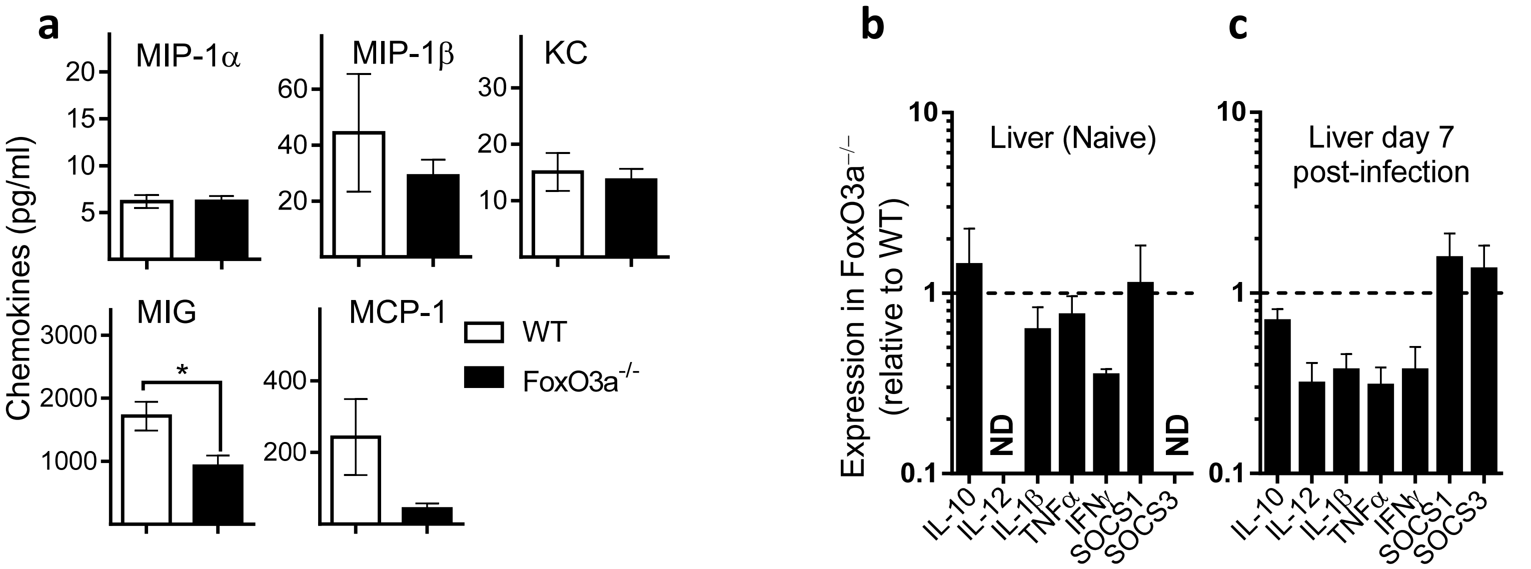

Supplementary Figure 1: Absence of FoxO3a is associated with reduced expression of inflammatory cytokines and chemokines in response to ST. a. Serum chemokine levels in WT and of FoxO3a$^{-/-}$ mice at day 7 post-infection with 10^4 ST-OVA (3 mice per group). All graphs depict mean ± SEM. (* $p< 0.05$). **b, c.** Expression of mRNA of various cytokines in the livers of FoxO3a$^{-/-}$ mice relative to WT mice, at day 0 (b) and day 7 (c) post-infection. Results are pooled from 3 mice (day 0) or 7-8 mice (day 7) per group. Graphs depict mean ± SEM. Data was analyzed using 2-tailed students t-test.

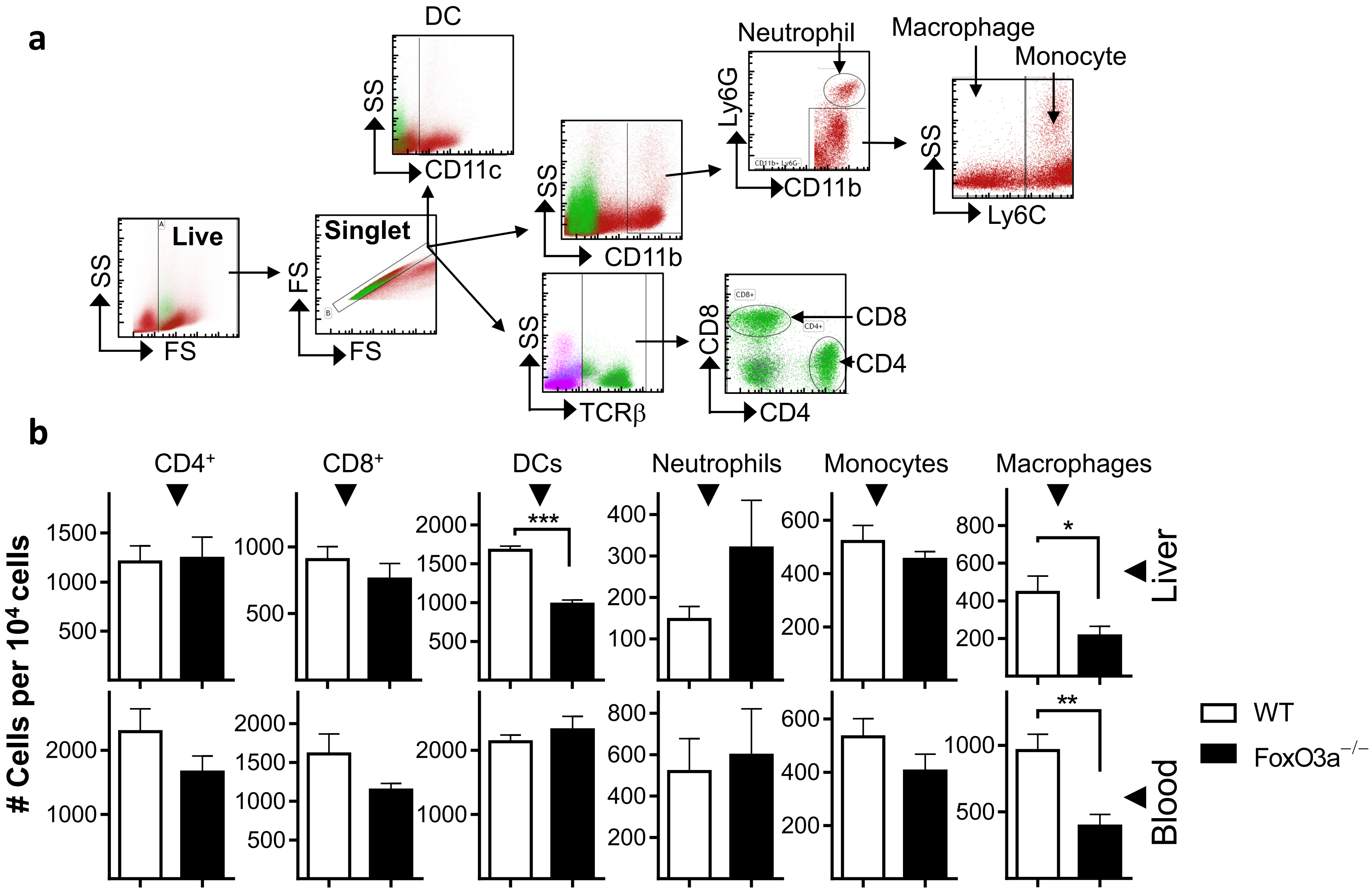

Supplementary Figure 2: FoxO3a deficiency leads to a reduction in the numbers of macrophages in the liver and blood during ST infection. a. Gating strategy used for identification of immune cell subsets. **b.** Numbers of CD4^{+} T cells, CD8^{+} T cells, dendritic cells, neutrophils, monocytes and macrophages in the liver (top, 8 mice per group) and blood (bottom, 4 mice per group) of WT and FoxO3a$^{-/-}$ mice at day 7 post-infection with ST-OVA. Results are pooled from at least three independent experiments with a minimum of 2-3 mice per group. Pooled data is shown. All graphs depict mean ± SEM. (* $p< 0.05$, ** $p<0.01$, *** $p<0.001$). Data was analyzed using 2-tailed students t-test.

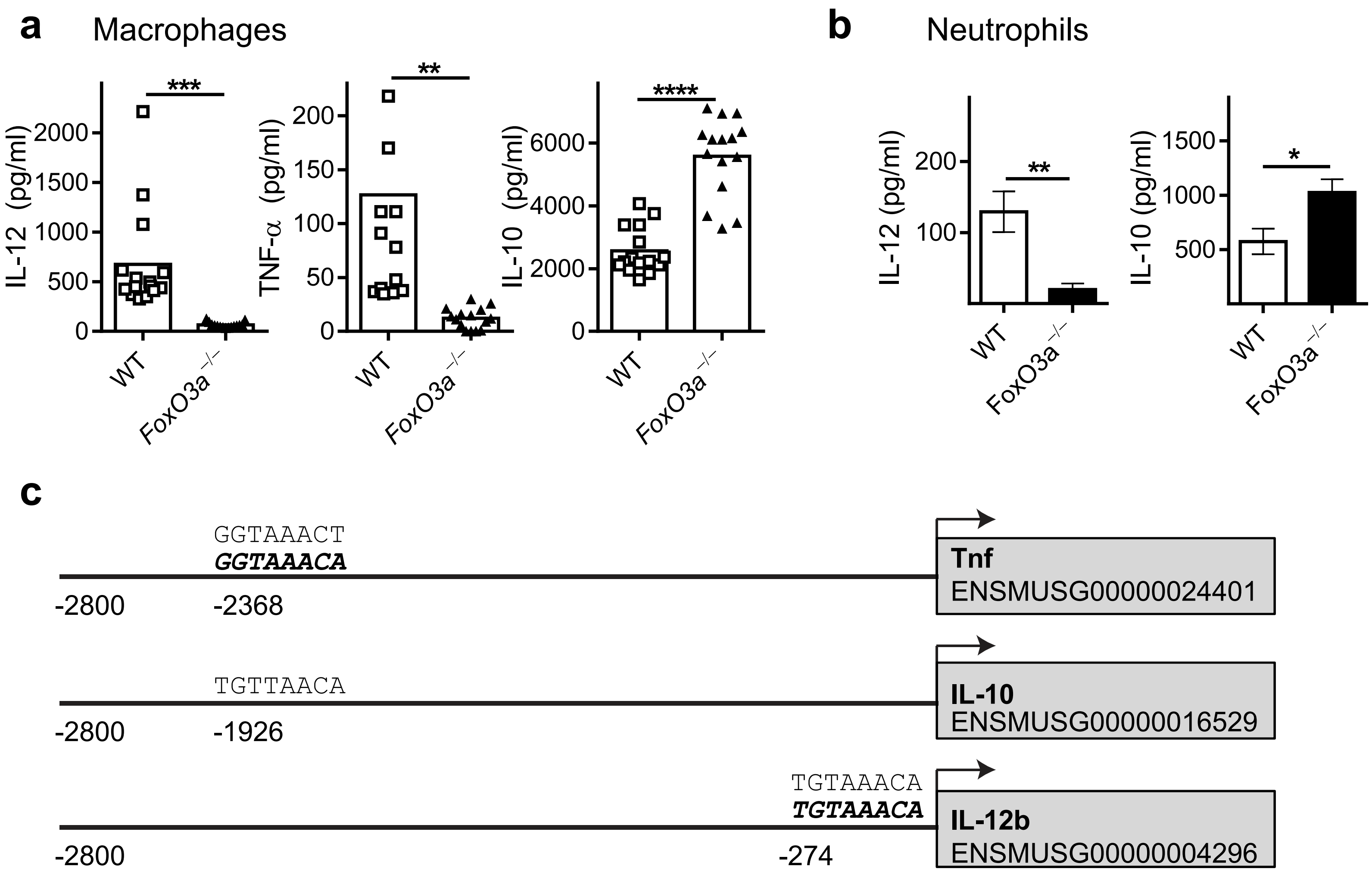

Supplementary Figure 3: FoxO3a deficiency impacts the balance between pro-inflammatory and anti-inflammatory cytokines in response to ST. a. TNF-α, IL-12 and IL-10 levels in the supernatants of WT and FoxO3a$^{-/-}$ macrophages infected with ST (10 MOI) at 18-24 hours post-infection. Results are pooled from at least three biological replicates with each being an average of three experimental replicates. All graphs depict mean ± SEM. (** $p< 0.01$, *** $p< 0.001$, **** $p<0.0001$). **b.** IL-12 and IL-10 levels in the supernatants of WT and FoxO3a$^{-/-}$ neutrophils infected with ST-OVA (10 MOI) at 18-24 hours post-infection. Results are pooled from four biological replicates with each being an average of three experimental replicates. All graphs depict mean ± SEM. (* $p< 0.05$, ** $p<0.01$). **c.** Predicted FoxO3a binding sites within the proximal promoters of genes deregulated in FoxO3a$^{-/-}$ cells are shown. Arrows represent the transcription start sites. Sequences in italics are the corresponding conserved FoxO3a motifs within the human promoter sequences. Data was analyzed using 2-tailed students t-test.

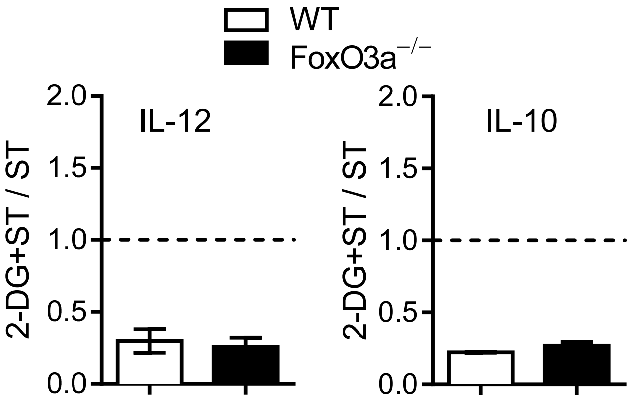

Supplementary Figure 4: Inhibition of glycolysis leads to a reduction in cytokine secretion. Ratio of IL-12 and IL-10 levels in 2-DG treated and infected macrophages over non-treated infected macrophages. Data is pooled from 2 independent experiments. Graphs depict mean ± SEM. Results are pooled from at least two independent experiments with a minimum of 2-3 mice per group.

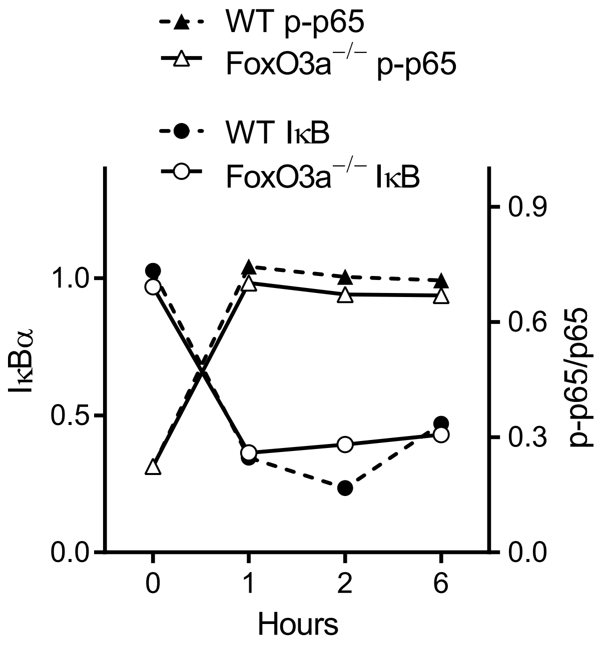

Supplementary Figure 5: FoxO3a deficiency does not impact p65 phosphorylation. Densitometry analyses of western blots (Fig. 6a) at various time intervals post-infection with ST-OVA (10 MOI). Results are pooled from at least two independent experiments with a minimum of 2-3 mice per group.

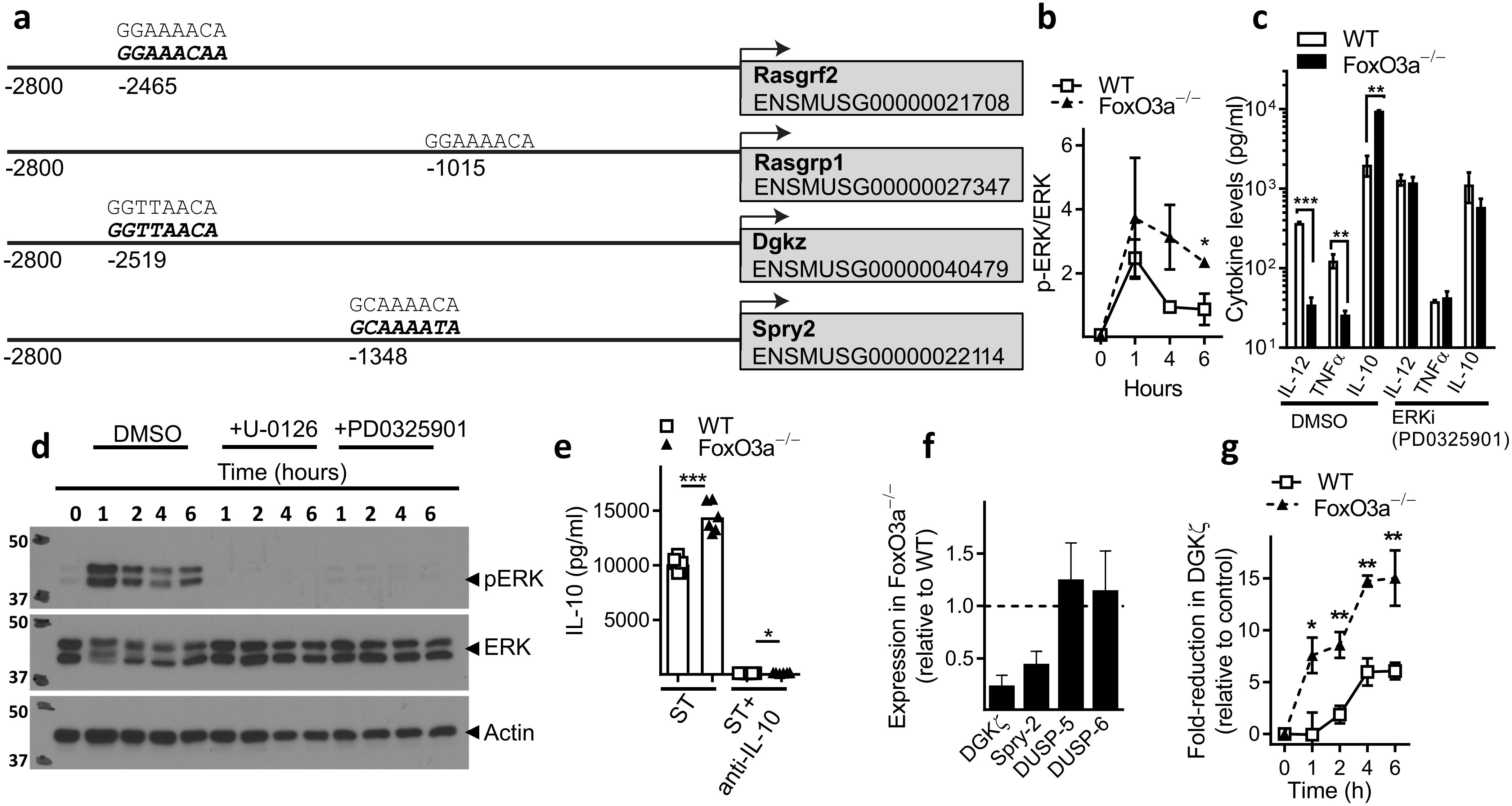

Supplementary Figure 6: Impact of FoxO3a on ERK signaling. a. Predicted FoxO3a binding sites within the proximal promoters of genes deregulated in FoxO3a$^{-/-}$ cells are shown. Arrows represent the transcription start sites. Sequences in italics are the corresponding conserved FoxO3a motifs within the human promoter sequences. **b.** Densitometry analyses of western blots for phospho-ERK and ERK expression in WT and FoxO3a$^{-/-}$ macrophages infected with ST-OVA (10 MOI). Data is pooled from two independent experiments. Graph depicts mean ± SEM. (* $p< 0.05$) **c.** Cytokine levels as measured in the supernatants of WT and FoxO3a$^{-/-}$ macrophages infected with ST-OVA (10 MOI) in the absence/presence of ERK inhibitor PD0325901 at 18-24 hours post-infection. Data is representative of at least two biological replicates with each being an average of 2-3 technical replicates. Graph depicts mean ± SEM. (** $p< 0.01$, *** $p< 0.001$). **d.** Western blot showing inhibition of phospho-ERK in macrophages infected with ST-OVA (10 MOI) in the presence of U-0126 and PD0325901.Data is representative of two independent experiments. **e.** IL-10 levels in the supernatants of WT and FoxO3a$^{-/-}$ macrophages infected with ST (10 MOI) in the presence of an anti-IL-10 antibody, at 18-24 hours post-treatment. Data is pooled from 2 independent experiments. Graph depicts mean ± SEM. (* $p< 0.05$, *** $p< 0.001$). **f.** Expression of mRNA of various genes in FoxO3a$^{-/-}$ macrophages relative to WT macrophages at 1 hour post-infection with ST-OVA (10 MOI). Results are pooled from four biological replicates per group. Graph depicts mean ± SEM. **g.** DGK-ζ mRNA levels in WT and FoxO3a$^{-/-}$ macrophages before and after infection with ST-OVA (10 MOI). Results are pooled from four biological replicates per group. Graph depicts mean ± SEM. (* $p< 0.05$, ** $p<0.01$). Data was analyzed using 2-tailed students t-test.

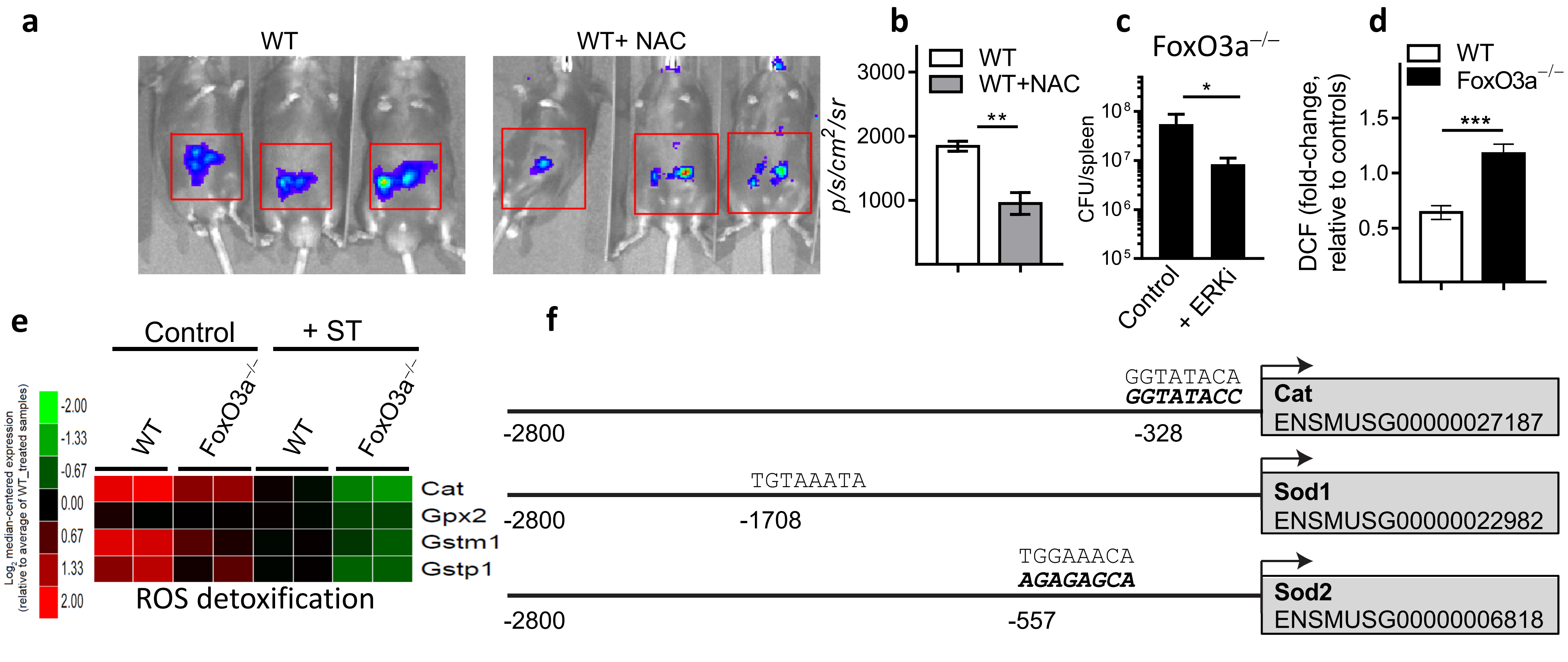

Supplementary Figure 7: FoxO3a is closely associated with reactive oxygen species levels. a & b. Reactive species levels in mice left untreated on treated with NAC at day 4 post-infection with ST-OVA (4 mice per group). Graph depicts mean ± SEM. **c.** Bacterial burden in the spleens of FoxO3a$^{-/-}$ mice left untreated or treated with ERKi over the course of infection (a minimum of 4 mice per group). **d.** Reactive species levels as measured by DCF staining in WT and FoxO3a$^{-/-}$ neutrophils following infection with ST-OVA (10 MOI). Data is pooled from 2 independent experiments. Graph depicts mean ± SEM. (*** p<0.001) **e.** Microarray data depicting the expression of genes involved in ROS detoxification that showed a change in expression of at least 50% in ST infected FoxO3a$^{-/-}$ macrophages as compared to ST infected WT macrophages (10 MOI) (2 mice per treatment). **f.** Predicted FoxO3a binding sites within the proximal promoters of genes deregulated in FoxO3a$^{-/-}$ cells are shown. Arrows represent the transcription start sites. Sequences in italics are the corresponding conserved FoxO3a motifs within the human promoter sequences. Results are pooled from at least two independent experiments with a minimum of 4 mice per group. Data was analyzed using 2-tailed students t-test.

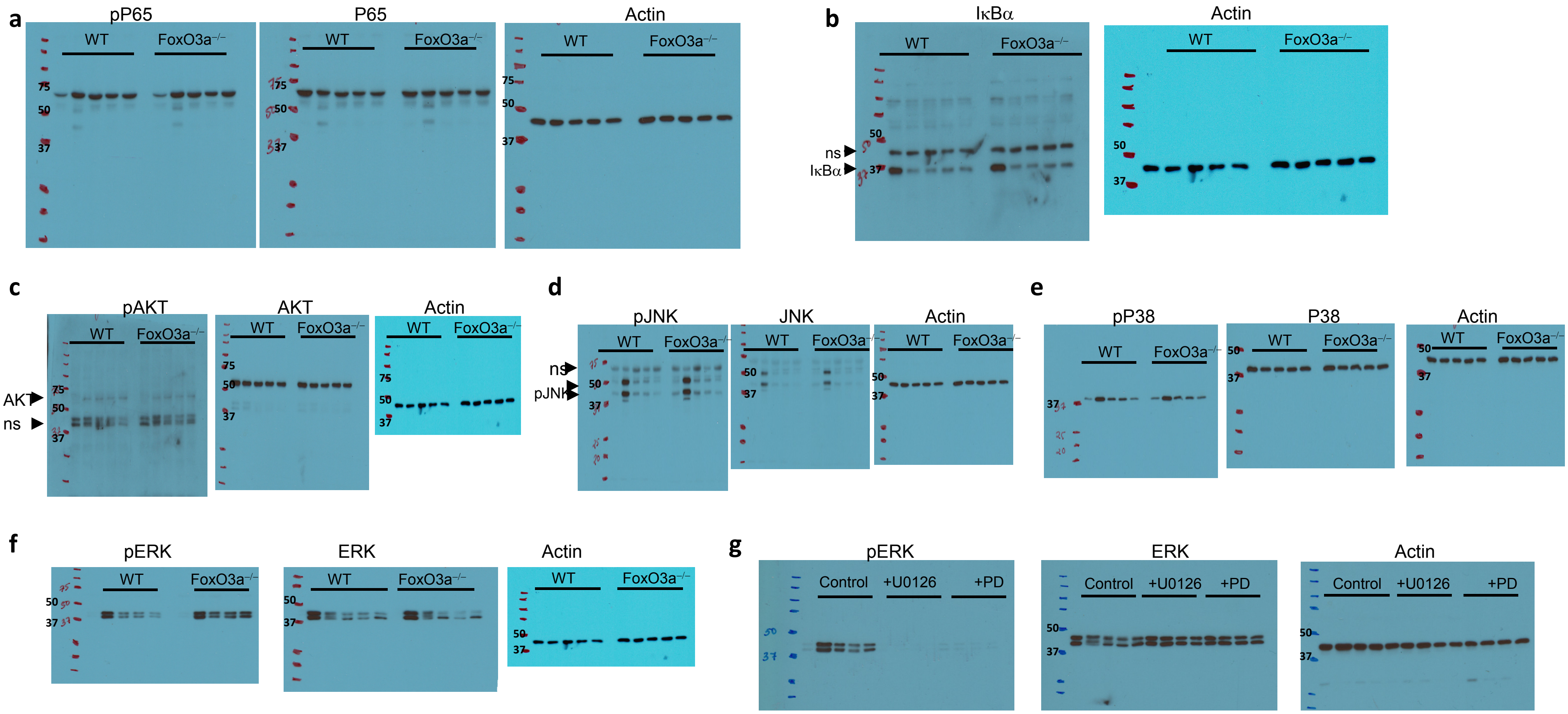

Supplementary Figure 8: Western blots of NF-κB, AKT, P38, JNK and ERK (a, b) Western blots of macrophages at various time intervals post-infection (indicated in Fig. 6) with ST-OVA (10 MOI) for expression of phospho-p65, p65 and IκBα. (c-e) Western blots of macrophages at various time intervals post-infection (indicated in Fig. 6) with ST-OVA (10 MOI) for the expression of pAKT, pJNK and pP38. (f, g) Western blots in macrophages at various time intervals post-infection (indicated in Fig. 7c) with ST-OVA (10 MOI) for the expression of pERK, ERK and JNK. Data is representative of two independent experiments.

Gene of interest	Forward primer (5′-3′)	Reverse primer (5′-3′)
Actin	GATCAAGATCATTGCTCCTCCTG	AGGGTGTAAAACGCAGCTCA
IL-10	GGTTGCCAAGCCTTATCGGA	GGGGAGAAATCGATGACAGC
IL-12	CCTTGCATCTGGCGTCTACA	AGGAGGTAGCGTGATTGACA
IL-1β	TGCCACCTTTTGACAGTGATG	TGATGTGCTGCTGCGAGATT
TNF	ACGTCGTAGCAAACCACCAA	ATAGCAAATCGGCTGACGGT
IFN-γ	CGGCACAGTCATTGAAAGCC	TGTCACCATCCTTTTGCCAGT
SOCS1	CCGCCAGATGAGCCCAC	TACCATCCTACTCGAGGGGC
SOCS3	TAGACTTCACGGCTGCCAAC	CGGGGAGCTAGTCCCGAA
SOD-1	GGAACCATCCACTTCGAGCA	CTGCACTGGTACAGCCTTGT
SOD-2	GTGTCTGTGGGAGTCCAAGG	AGCGGAATAAGGCCTGTTGT
Catalase	GTGCCCCCAACTATTACCCC	AGAATGTCCGCACCTGAGTG
GADD45A	TGGTGACGAACCCACATTCA	CGGGAGATTAATCACGGGCA
DGKζ	CGGCTGCCTGGTGTAGACA	GCACCTCCAGAGATCCTTGATG
Spry-2	TCCACCGATTGCTTGGAAGT	ACACATCTGAACTCCGTGATCG
DUSP-5	GCACCACCCACCTACACTAC	CCTTCTTCCCTGACACAGTCAAT
DUSP-6	GTTCTACCTGGAAGGTGGCT	TCCGTTGCACTATTGGGGTC
HIF1-α	TGACGGCGACATGGTTTACA	ACTGGGCCATTTCTGTGTGT
iNOS	GCCACCAACAATGGCAACAT	TCGATGCACAACTGGGTGAA
Mannose Receptor	GGCTGATTACGAGCAGTGGA	ATGCCAGGGTCACCTTTCAG
Arginase 1	TTTTAGGGTTACGGCCGGTG	CCTCGAGGCTGTCCTTTTGA
AMPKα1	GCCATGCGCAGACTCAGTTC	ACTCGTGCTTGCCCACCTT
TGF-β	TGACGTCACTGGAGTTGTACGG	GGTTCATGTCATGGATGGTGC

Supplementary Table 1
List of primer sequences
